# Genetic architecture of low‐temperature germinability in rice revealed by integrated maps of SNPs and structural variation

**DOI:** 10.1111/tpj.71184

**Published:** 2026-10-27

**Authors:** Youshuai Shi, Kai Xu, Yamei Lv, Xiaoyu He, Tingting Yu, Fengcai Wu, Lunping Liang, Ting Feng, Liang Zhang, Erbao Liu, Zhikang Li, Wensheng Wang, Min Li, Chaopu Zhang

**Affiliations:** ^1^ College of Agronomy Anhui Agricultural University Hefei China; ^2^ State Key Laboratory of Crop Gene Resources and Breeding Beijing 100081 China; ^3^ Chinese Academy of Agricultural Sciences Institute of Crop Sciences Beijing 100081 China

**Keywords:** low‐temperature germinability, structural variation, divergent region, *Xian–Geng* differentiation, machine learning

## Abstract

Low‐temperature germinability (LTG) limits rice direct seeding, yet its genetic architecture and regulatory roles of structural variations (SVs) remain largely unclear. We evaluated LTG in 403 rice accessions at four temperatures (13°C, 15°C, 18°C, 21°C). Integrating SNP/SV–GWAS, RNA‐seq, gene editing, population genetics, and machine learning, we dissected the genetic basis of LTG and built a genomic selection (GS) prediction model. The panel showed extensive LTG variation, which was negatively correlated with origin latitude. SV‐ and SNP‐based GWAS for LTG detected 119 and 127 QTLs, respectively, with 25 loci overlapping between the two datasets. Combining GWAS and RNA‐seq, we screened 30 candidate genes and 48 favorable haplotypes, with high‐LTG favorable alleles enriched in low‐latitude *Xian* (*Indica*) accessions. We identified a deletion in the *qLTG3‐1* coding region that negatively regulated LTG, and gene editing (CRISPR/Cas9) confirmed *qLT11d* is a key gene for LTG. Population genetic analysis identified 29 inter‐subspecies (*Xian–Geng*) and 37 intra‐*Xian* divergent regions for LTG, implying shared selected regions across and within subspecies under low temperature. Using SNP and SV data, we built corresponding genomic selection (GS) models with the machine learning‐based MFGP method, achieving high prediction accuracy across all temperatures. All cross‐temperature prediction correlations exceeded 0.55, with optimal performance at 18°C. Our results clarify rice LTG genetic architecture and suggest potential regulatory roles for SVs that merit further investigation, providing candidate genes, favorable haplotypes, and a GS model for LTG genetic improvement in direct seeding rice.

## Significance Statement

Low‐temperature germinability (LTG) is critical for direct seeding rice, but its genetic basis remains poorly understood. By integrating SNP and structural variation (SV) maps, we uncovered the LTG genetic architecture, identified 30 candidate genes with favorable haplotypes, and suggested potential regulatory roles for SVs that merit further investigation. A machine learning‐based genomic selection model achieved high prediction accuracy, providing valuable resources for breeding climate‐resilient rice varieties.

## INTRODUCTION

In the world rice production, direct seeding is increasingly popular in recent years compared with the traditional transplanting method because of its easiness and lower labor costs (Jiang et al., 2006; Mahender et al., 2015). The optimum temperature range for rice seed germination is approximately from 25°C to 35°C. However, soil temperatures during rice sowing are frequently below 18°C and even below 15°C for early rice crops in most areas of south China or in high‐latitude regions (Fujino et al., 2004). Poor seed germination and seedling establishment caused by low temperature have become one of the major factors limiting the application of direct seeding in rice (Yang et al., 2020). Thus, rice varieties with high low‐temperature germinability (LTG) are needed for direct seeding rice because LTG can not only ensure high rice germination rate and uniformity but also increase its ability to compete against weeds in the field (Li, Dong, et al., 2023). The two major rice subspecies, *Xian/Indica* and *Geng/Japonica*, are known to show considerable differences in LTG. However, conflicting results were reported from different studies regarding the difference in LTG between *Xian* and *Geng*. For example, a study reported that most *Xian* varieties tended to show significantly higher LTG than *Geng* and *Aus* accessions (Yang et al., 2020), even though most *Geng* accessions were more tolerant to cold than *Xian* accessions at the germination stage (Wang, Li, et al., 2023). In a separate study, no significant difference in LTG was found between *Xian* and *Geng* subspecies (Baruah et al., 2009).

Recently, considerable efforts have been taken to identify QTL affecting LTG using natural or bi‐parental mapping populations in rice. As of now, at least nine LTG genes (*qLTG3‐1*, *OsSAP16*, *OsCAT2*, *OsABI5*, *OsD2*, *OsMYB30*, *OsTPP1*, *OsSnRK1.1*, and *OsUBC12*) have been cloned and characterized in rice (Fujino et al., 2008; Kim et al., 2022; Li et al., 2021; Li, He, et al., 2023; Wang, He, et al., 2023; Yu et al., 2024). Of these, *qLTG3‐1* encoding a protein of protease inhibitor/seed storage/LTP family protein precursor function was the first reported QTL gene controlling LTG in rice (Fujino et al., 2008). *OsSAP16* encoding a zinc‐finger domain protein has been demonstrated as a causal gene for one major LTG QTL, and its varied expression contributed to LTG variation (Wang, Li, et al., 2023). Both *OsTPP1* and *OsMYB30* controlled rice cold tolerance. Low temperature resulted in greatly enhanced expression of *OsMYB30* and *OsTPP1* and increased trehalose content in rice seedlings, but inhibited rice seed germination (Yu et al., 2024). Similar to most rice traits, the reported QTLs/genes only account for a small portion of genes underlying LTG of rice, and the obtained information is insufficient to facilitate marker‐assisted breeding to improve LTG of rice.

As an important type of genomic variations, structural variations (SVs) have been widely found across rice genomes. Due to their relatively large sizes, SVs are expected to have large phenotypic effects when occurring in either gene coding sequences or promoter regions (Li, He, et al., 2023; Wang, Li, et al., 2023). Thus, SVs are powerful markers in detecting genes affecting complex traits using GWAS. There are two reported cases in which genes with large effects on rice LTG were identified, including *qLTG3‐1* caused by a deletion in its coding region (Fujino et al., 2008), and *OsUBC12* at which increased expression led to increased LTG from a transposon insertion in its promoter (Zhang et al., 2024). Nevertheless, there were relatively few reported cases where SVs were used for large‐scale gene/QTL discovery.

Here, we reported an effort to genetically dissect rice LTG using GWAS of next‐generation sequencing (NGS)‐based detection of single‐nucleotide polymorphisms (SNPs) and SVs and comprehensive phenotyping of 403 well‐selected rice panel representing rice accessions from major rice populations worldwide. Large numbers of SNPs and SVs significantly associated with LTG were identified, which revealed the genetic architecture of rice LTG regarding its subspecific differentiation, rich within subspecies/population diversity and interaction with different low‐temperature regimes. Moreover, candidate genes and favorable alleles for important LTG QTLs were prioritized using a combined strategy of multi‐omic and phenotypic data. Our results provide insights into adaptive mechanism(s) underlying LTG variation during rice evolution and important information to facilitate genetic improvement of rice LTG using molecular breeding strategies.

## RESULTS

### Population structure of the 403 rice accessions

The 403 rice accessions from 26 countries were used for LTG analysis. These accessions were sequenced with an average sequencing depth ~16.8×, and 96.3% of them had sequencing depths greater than 10× (Table S1). After filtering out low‐quality SNPs with minor allele frequency (MAF) ≤0.05 and missing rate ≥20%, a total of 3.2 M SNPs for the whole panel were identified (Figure S1A). We then constructed a phylogenetic NJ tree using the SNP map, which classified the 403 accessions into five major subgroups, including 259 *Xian*, 121 *Geng*, nine admixtures (*Adm*), four *Aus*, and eight *Basmati* (*Bas*) accessions (Figure 1A, E; Table S1). PCA revealed that the first PC accounted for 36.4% of the total SNP genetic variance and separated the two major subspecies *Xian* and *Geng*, while the second PC explained 3.8% of the total SNP genetic variance and separated the *Aus* accessions from the *Xian* and *Geng* accessions (Figure 1B).

**Figure 1:**
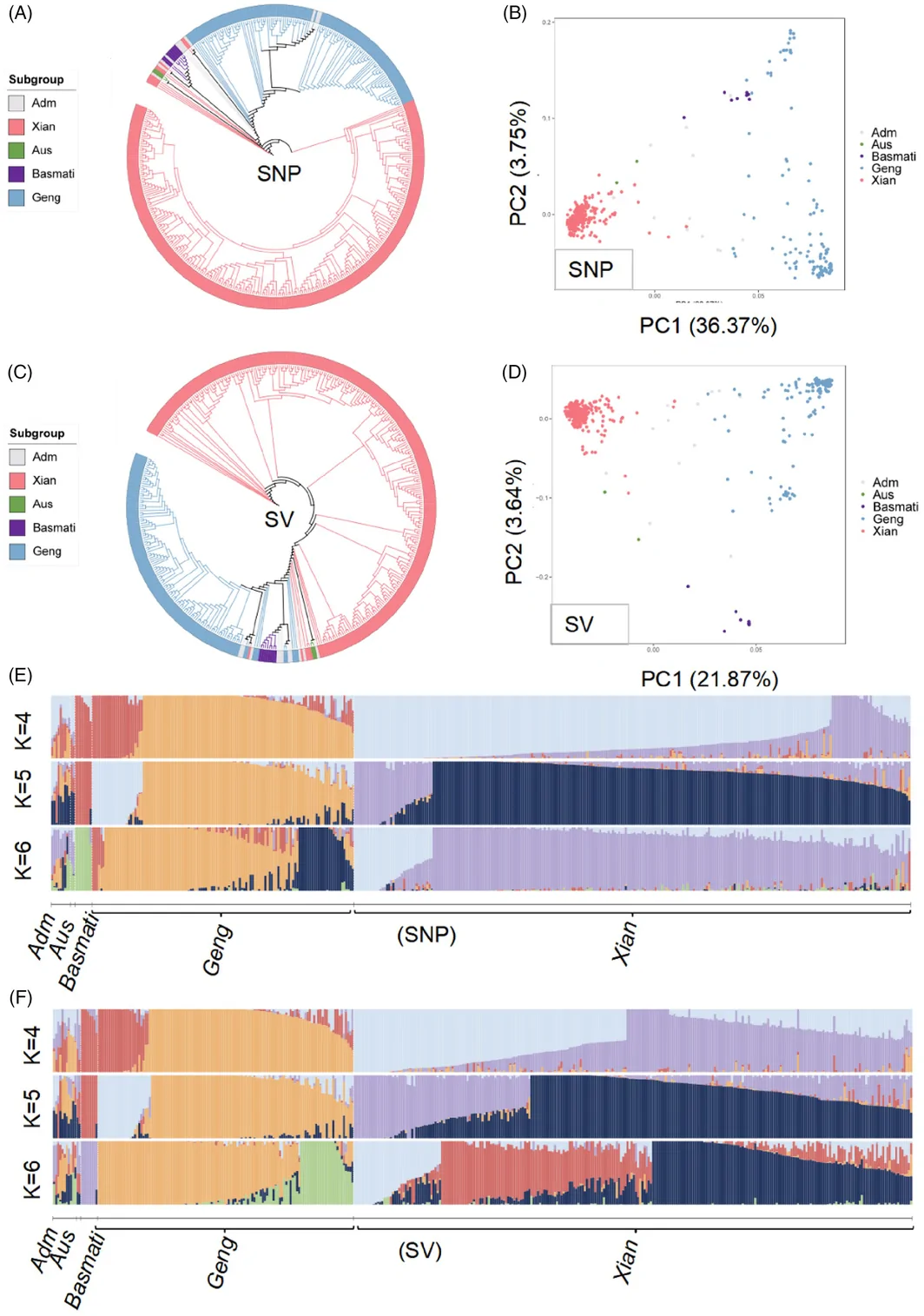
Genetic structure and population differentiation of 403 rice accessions. (A, C) Neighbor‐joining (NJ) trees constructed based on all single‐nucleotide polymorphisms (SNPs) (A) and structural variants (SVs) (C) in the whole panel, respectively. (B, D) Principle component (PC) analysis for 403 rice accessions based on the SNPs (B) and SVs (D). PC1 and PC2 refer to the first and second principal components. (E–F) Population structure of 403 rice accessions determined based on the SNPs (E) and SVs (F). The *y*‐axis quantifies cluster membership, and the *x*‐axis lists the different accessions.

A total of 67 134 high‐quality SVs were identified in the whole panel after filtering out low‐quality SVs (Figure S1B). The SV map included 2759 duplications, 40 792 deletions, 2269 inversions, 76 insertions, and 21 238 break‐ends (BNDs) (Table S3). These SVs were distributed on all 12 chromosomes, with an average of 5595 SVs in each chromosome. Based on the SV map, the 403 rice accessions could be divided into two major subpopulations, including 259 *Xian* and 121 *Geng* accessions, and three subgroups of 11 *Adm*, four *Aus*, and eight *Bas* accessions (Figure 1C, F). PCA based on SVs yielded a similar pattern to SNP results (Figure 1D). PC1 and PC2 accounted for 21.9 and 3.6% of total SV genetic variance, respectively, which classified the 403 accessions into five subgroups (*Xian*, *Geng*, *Adm*, *Bas*, and *Aus*). The subgroup classification based on SNPs was highly consistent (99.3%) with that based on SVs (only four *Xian* or *Geng* accessions were mixed with the *Adm* subgroup). Moreover, the results of SNP‐ and SV‐based subgroup classification were also highly consistent (98.8%) with the 3 K results (Wang et al., 2018) among the overlapped accessions (Table S1). Two *Xian* accessions from south Asia in 3 K results were misclassified into *Aus* subgroup based on the SNP and SV maps. Thus, these two accessions were most likely derived from the intercross between *Aus* and South Asia *Xian* subpopulation.

In SV detection, we identified three genotypes in the whole, *Xian*, and *Geng* panels, including the ATL allele (accessions possessing the homozygous alternative allele), Ref (accessions with the homozygous reference Nipponbare type of allele, NSV), and several Het (accessions possessing the heterozygous alternative allele). We noted that most SVs had low frequencies of NSVs in the 403 accessions, while several genome regions had extremely low frequencies of SVs (Figures S2, S3). Of all SVs identified, 14 797 SVs were present only in panel *Xian* but absent in panel *Geng*, and thus named as *Xian*‐specific SVs (Figure S4). In panel *Xian*, most (72.8%) of these *Xian*‐specific SVs had low frequencies <5%, and 3330 of them had frequencies >5% with only 53 SVs had frequencies >50%. Similarly, we identified 5574 *Geng*‐specific SVs (absent in panel *Xian*). Most (67.2%) of these *Geng*‐specific SVs had low frequencies <5% in panel *Geng*, while 1829 of them were present in >5% of *Geng* accessions with 62 common SVs present in >50% the *Geng* accessions (Figure S4; see also Figures S2 and S3 for genome‐wide SV frequency distributions). Thus, the subspecies‐specific SVs account for 30.3% of the SVs identified in this study and contributed greatly to the *Xian–Geng* subspecific differentiation.

### Phenotypic variations of 403 rice accessions for the LTG parameters

The 403 rice accessions showed considerable variations for the measured seed germination and seedling rates, and the calculated LTG parameters under the four temperature treatments. As expected, the time required for germination increased significantly as the testing temperatures decreased (Figure S5), and so did the germination index (GI) and seedling index (SI) in the assessed accessions (Table S4). The average time required to reach 50% seed germination (GT50) was 201.7 h at T13, 142.2 h at T15, 101.5 h at T18, and reduced to 76.3 h at T21 (Figure S5). Clearly, the low temperatures could progressively delay seed germination and reduce early seedling rates. Analysis of variance (ANOVA) revealed significant differences between *Xian* and *Geng* subspecies, among different accessions within panel *Xian* or *Geng*, and among the four temperatures. Moreover, significant interactions were found between the subspecies and temperatures for the assayed parameters (Table S5), suggesting that both population genetic and environmental factors affected LTG. On average, the *Geng* accessions showed significantly delayed germination and early seedling growth compared with the *Xian* accessions across the four low‐temperature treatments (Figure S6).

### QTL for LTG using SNP‐based GWAS

Because of large phenotypic variations for the measured LTG parameters present within the *Xian* and *Geng* subspecies (Table S4), GWAS was performed on the whole panel as well on panels *Xian* (*n* = 259) and *Geng* (*n* = 121). After filtering out low‐quality SNPs, three SNP datasets, including 3.2 M SNPs for panel whole, 2.6 M SNPs identified for panel *Geng*, and 3.0 M SNPs for panel *Xian*, were obtained. All the phenotype datasets were used to perform GWAS in panels whole, *Xian*, and *Geng*, respectively. A total of 506 SNPs were preliminarily detected to be significantly associated with one or more LTG parameters across the four temperatures in the three panels. These included 269 SNPs in panel whole, 212 in panel *Xian*, and 25 in panel *Geng*. Among these significant SNPs, 75 were associated with three or more parameters, and eight were associated with 10 or more parameters. Because multiple highly linked SNPs all significant in a small region could be attributed to a single QTL, we merged those SNPs identified by multiple parameters, temperatures, and panels, which led us to identify 127 QTL for LTG based on SNP–GWAS (Figure 2A; Table S6), including 90 QTL in panel whole, 63 QTL in panel *Xian*, and 19 QTL in panel *Geng*, respectively. Of the QTL identified, 30 QTL were shared by panels whole and *Xian*, 12 by panels whole and *Geng*, seven by panels *Xian* and *Geng*, and four by all three panels (Figure 2C). For the temperature treatments, 58 QTL were identified in T13, 64 QTL in T15, 71 QTL in T18, and 62 QTL in T21. Among them, 36 QTL were identified in two temperatures, 25 QTL were detected in three of the temperatures, and 13 QTL were simultaneously identified in all four temperatures (Figure 2D).

**Figure 2:**
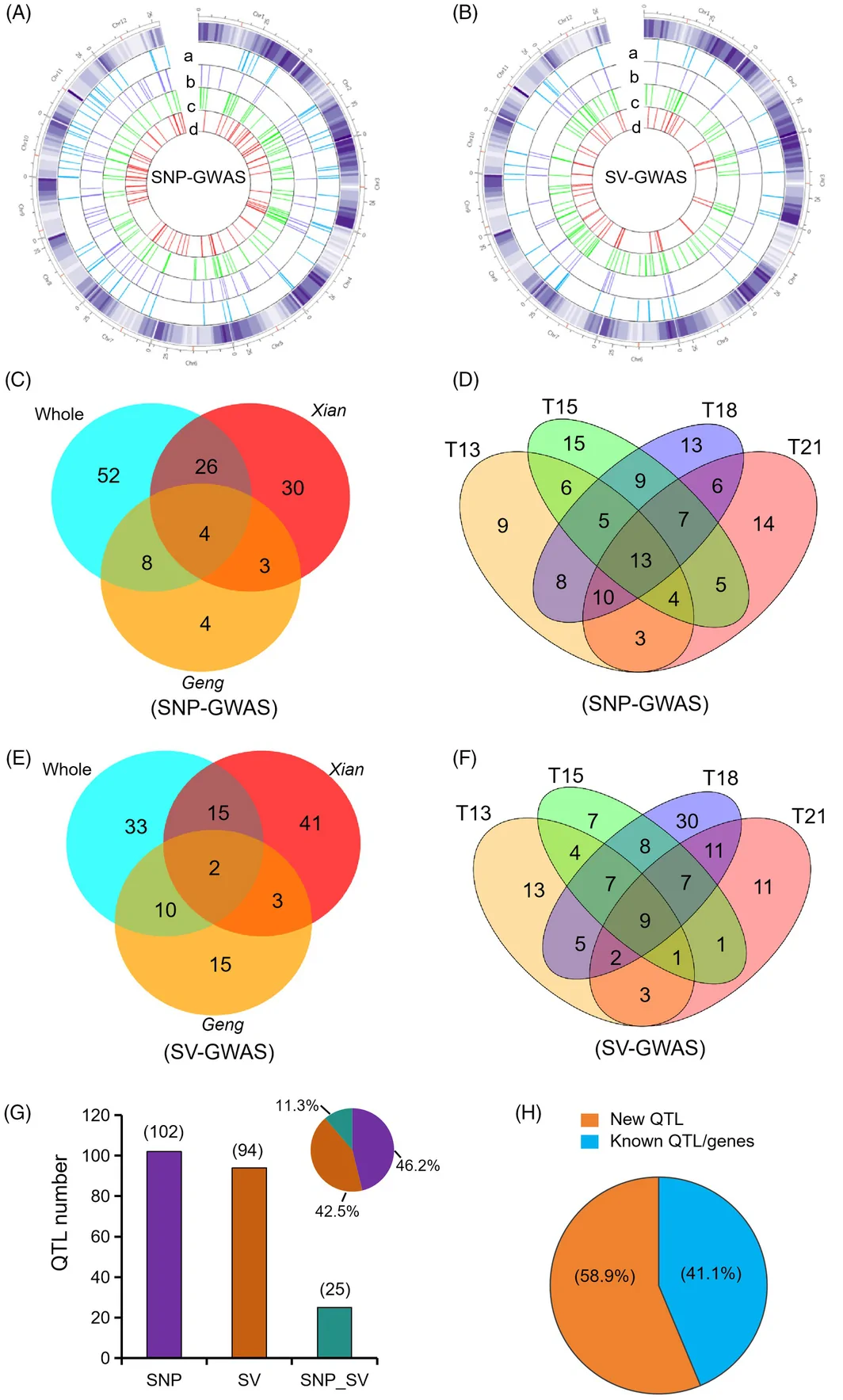
Summary of quantitative trait locus (QTL) for low‐temperature germination (LTG) detected based on the SNP–GWAS and SV–GWAS. (A, B) Distribution of the QTLs identified by SNPs (A) and SVs (B) in the four temperatures. The outer to the inner circles represent the QTLs identified in T13 (a), T15 (b), T18 (c), and T21 (d). The QTLs identified for LTG using SNP (C, D) and SV (E, F) datasets in the whole, *Xian*, and *Geng* panels. (G) Number of QTLs for LTG detected by different categories of markers. SNP and SV indicate the QTLs that could be only identified by SNP or SV‐based GWAS, respectively; SV_SNP indicates the QTLs detected by both SV‐ and SNP‐based GWAS. (H) The ratios for LTG linked with known QTL/genes related to LTG in rice through GWAS and linkage mapping analyses.

### LTG QTL identified by SV‐based GWAS

To identify the SV‐associated LTG, three SV datasets, including 67 134 SVs for the whole panel, 46 147 SVs for panel *Xian*, and 39 167 SVs for panel *Geng*, were used in GWAS after filtering out low‐quality SVs. Together, 566 SVs were preliminarily detected to be significantly associated with LTG in the three panels (Figure 2B; Table S7a). Of these, 57 SVs were significantly associated with three or more parameters and 12 SVs were detected by ten or more parameters. After merging the SVs identified by multiple parameters, different panels and temperatures, a total of 119 QTL were identified for LTG in the four temperatures (Table S7b). Specifically, 60 QTL were detected in the whole panel, 61 QTL in panel *Xian*, and 30 QTL in panel *Geng*, respectively. Among them, 15 QTL were commonly detected in panels whole and *Xian*, 10 in panels whole and *Geng*, and three in panels *Xian* and *Geng* (Figure 2E). For each temperature, 44 QTL were identified in T13, 44 QTL in T15, 79 QTL in T18, and 45 QTL in T21, respectively. Among them, 32 QTL were commonly identified in two temperatures, 17 QTL were detected in three temperatures, and nine QTL were identified across all four temperatures (Figure 2F).

By comparing the results from SNPs and SVs, 102 QTL (46.2%) were exclusively identified by SNPs, 94 (42.5%) QTL were detected only by SVs, and 25 (11.3%) QTL had overlapping peaks between SNP‐based and SV‐based GWAS when applying the population‐specific LD decay distance of approximately 220 kb (Figure 2G). Two pairs, *qLT6a/qLTv6a* and *qLT8e/qLTv8g*, had both lead variants located within the same annotated gene or its immediate flanking region. For example, the lead SNP and lead SV of *qLT8e/qLTv8g* are both located within Os08g0313600. Among the overlapping QTL, 10 could be identified in two of the temperatures, 7 QTL in three of the temperatures, and 7 QTL in all four temperatures (Figure S7). These results indicated that the SV‐based GWAS was an important complement to the conventional SNP‐based GWAS.

### Important QTL for LTG

Among the identified LTG QTL, many co‐localize in or near the same regions by all four temperatures, multiple parameters, and/or multiple panels. These QTL associated with multiple seedling and germination parameters in four temperatures were thus considered important LTG QTL. Based on these criteria, 13 important QTL for LTG were identified by SNP–GWAS (Table S6; summarized in Table S8 for important ones). Additional eight QTL (*qLT1i*, *qLT2n*, *qLT4c*, *qLT7j*, *qLT8a*, *qLT9e*, *qLT10g*, and *qLT11b*) were commonly detected in previous studies through GWAS or linkage mapping. For SV–GWAS, nine important QTL were found (Table S6; summarized in Table S8 for important ones). Of them, four QTL (*qLTv4k*, *qLTv5i*, *qLTv7g*, and *qLTv11l*) were detected in panels whole and *Xian*, *qLTv3*a was identified in panels whole and *Geng*, and four (*qLTv3a*, *qLTv6a*, *qLTv7g*, and *qLTv8a*) were previously reported. Thus, these major QTL were further investigated to dissect the genetic effects on LTG.

### Haplotype analysis of *qLTG3‐1* and adaptation to LTG

A major QTL, designated *qLT3a* (QTL for low‐temperature germinability (LTG), chromosome 3a, detected by SNP–GWAS) and *qLTv3a* (the corresponding SV–GWAS‐detected locus), located at approximately 0.22 Mb on chromosome 3, was detected by SV‐based GWAS in panels whole and *Geng* across four temperatures. This QTL was also simultaneously detected by SNP‐based GWAS in panels whole and *Xian* in three temperatures (Tables S6–S8). The lead SV (SV_3_220111, Del) in the QTL region locates in the coding region of Os03g0103300 or *qLTG3‐1* (Figure 3A–D). *qLTG3‐1* was reportedly affecting LTG, resulting from a 71‐bp deletion (Zhang et al., 2024). Interestingly, the lead SV (SV_3_220111, Del) locates in the same region harboring the 71‐bp deletion (Figure 3G). All rice accessions carrying the corresponding deletion showed a significantly decreased seed germination in panels whole and *Geng* (Figure S8) as compared with the accessions carrying NSV, suggesting that the SV exhibited a negative effect on LTG. This SV had a low frequency of 8.4% (34/403) in the rice accessions, 31 of which were present in panel *Geng* and only one in *Xian* (Figure 3E). Detailed examination of the SV in 356 rice genomes by the long reads technology (Wang, He, et al., 2023) revealed a similar result that this deletion was present only in 10 *Geng* accessions and one wild rice accession (Figure 3F).

**Figure 3:**
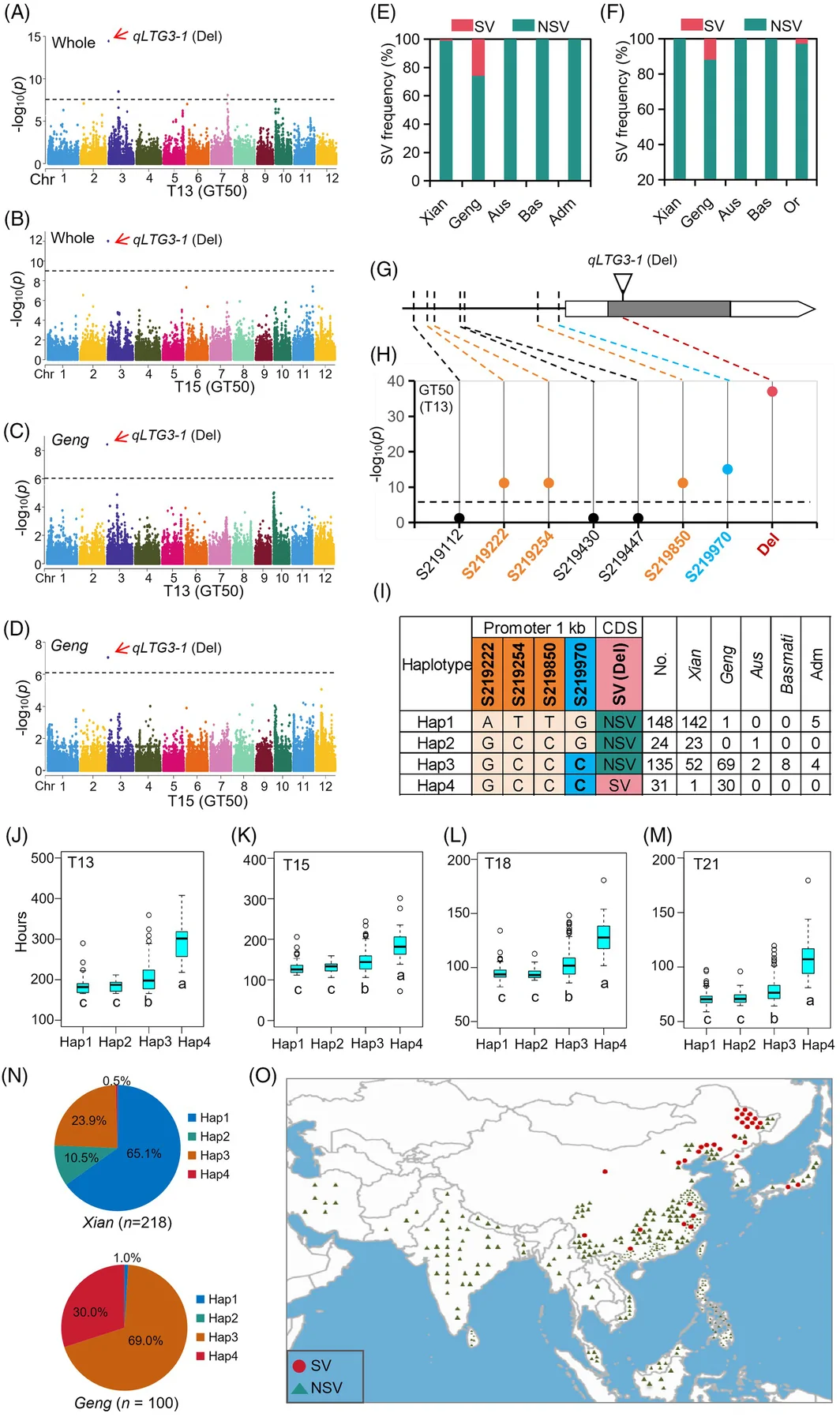
Natural variation analysis in the promoter and coding region of Os03g0103300 (*qLTG3‐1*). (A–D) Manhattan plots of GT50 parameter (the time (hours) required to reach 50% germination percentage of the accession) surrounding the SV peak on chromosome 3 in the whole (A, B) and *Geng* (C, D) panels. The red arrow indicates the position of lead SV (a Del). (E) Distribution of the accessions carrying SV and NSV genotypes in different subpopulations of 403 rice accessions in this study. (F) Distribution of the accessions carrying SV and NSV genotypes in different subpopulations of 365 rice accessions with long reads sequencing technology from database of rice variation map (RSPVM) (Wang, He, et al., 2023). The red and green represent SV and NSV ratios, respectively. (G) Gene structure and DNA polymorphism in Os03g0103300 gene. The vertical lines indicate seven high‐quality SNPs (after removing the missing genotype in more than 20 accessions) in Os03g0103300 gene promoter region. (H) Gene‐based GWAS (promoter region and coding region) for LTG. Horizontal dashed black line corresponds to a reference threshold (−log_10_(*P*) >5.0). The red dot denotes the peak SV (a Del); the three orange and a blue (peak SNP) dots indicate four major SNPs that are significantly associated with LTG. (I) Haplotype analysis of Os03g0103300 based on the four major SNPs in the gene promoter and the SV in coding region. The accessions carrying any one of the missing genotype were ignored. (J–M) GT50 of four haplotypes of Os03g0103300. Different letters denote significant difference according to the Duncan test at *P* < 0.05. (N) Distribution of four haplotypes in *Xian* and *Geng* subpopulations. (O) Geographic distribution of the rice accessions used in this study. The accession carrying the SV and NSV is displayed as a red dot and green triangle, respectively.

To clarify whether SNPs in Os03g0103300 region also affect LTG, we examined the sequences of Os03g0103300 across all rice accessions and found no non‐synonymous SNPs in its coding region, except for seven high‐quality SNPs in its promoter region (Figure 3G). A further gene‐based significance test revealed that only four SNPs (S219222, S219254, S219850, and S219970) and SV_3_220111 (*P* = 9.0 × 10^−38^) were significantly associated with the LTG parameters (Figure 3H). Among the four SNPs, S219970 exhibited the largest effect on LTG (*P* = 8.7 × 10^−16^), while the remaining three SNPs showed the same effects (*P* = 6.5 × 10^−12^) on LTG. The haplotype analysis combining the four SNPs and SV revealed four major haplotypes in 338 accessions (removing those accessions carrying missing genotype and rare haplotypes) (Figure 3I). Significant differences in the parameters (GT50) assayed in all four temperatures were detected among the four major haplotypes (Figure 3J–M). The accessions with Hap1 and Hap2 exhibited similarly higher LTG levels than those carrying Hap3 and Hap4, indicating that Hap1 and Hap2 were favorable alleles for LTG, while the accessions carrying Hap4 exhibited the lowest LTG level. These results suggested that SNPs S219222, S219254, and S219850 in the promoter had no effects on LTG. However, direct comparison between Hap2 and Hap3 suggested that the change of S219970^*G*^ to S219970^*C*^ significantly decreased LTG, indicating S219970 is a candidate SNP that warrants further functional validation (Figure 3J–M). This could explain why accessions carrying Hap4 S219970^*C*^ and SV_3_220111 (Del) alleles showed the lowest LTG.

We noted that 65.1% of the *Xian* accessions carried the favorable Hap1, and only 0.5% of them carried disadvantageous Hap4. The opposite was true in panel *Geng*, with only 1.0% *Geng accessions carrying* the favorable Hap1, but 99.0% of them carried the disadvantageous Hap3 or Hap4 (Figure 3N). Interestingly, most of the 403 rice accessions distributed in the high‐latitude regions carried the deletion (Figure 3O), while most of the rice accessions from low‐latitude regions did not have the deletion and exhibited higher levels of LTG. To visualize the geographic distribution of the four qLTG3‐1 haplotypes (Hap1–Hap4), we mapped them across the 403 accessions (Figure S9). Mirroring the deletion allele pattern (Figure 3O), the low‐LTG Hap4 was enriched in high‐latitude *Geng* accessions, whereas high‐LTG Hap1 and Hap2 were prevalent in low‐latitude *Xian* accessions, further supporting the role of *qLTG3‐1* in latitudinal adaptation.

### Comparative transcriptome analysis of the response to LTG

To compare the transcriptomic responses to low temperature between high‐ and low‐LTG accessions, eight extreme‐phenotype accessions, including four high‐LTG accessions (two *Xian* accessions: YH72 and CA; two *Geng* accessions: GM2 and HD502) and four low‐LTG accessions (two *Xian* accessions: TP3 and F88; two *Geng* accessions: SG5 and NG47), were selected for RNA‐seq analyses. The four high‐LTG accessions exhibited significantly higher seed germination ability than the four low‐LTG accessions under all four temperatures (Figure 4A, B). Based on the criteria of |log_2_FoldChange| ≥ 1.0 and *P*‐value <0.05, the total numbers of DEGs in the four low‐LTG accessions were 3530 and 3396 for TP3, 2930 and 3921 DEGs for F88, 3328 and 5673 DEGs for SG5, 3279 and 3963 DEGs for NG47 after 4 h (CT4) and 8 h (CT8) cold treatments, respectively (Figure 4C, D). Of these DEGs, 552 and 734 were common ones in the four low‐LTG accessions at CT4 and CT8, respectively (Figure 4E, F). The total numbers of DEGs detected in the four high‐LTG accessions were 4928 and 5847 for GM2, 4113 and 5924 DEGs for HD502, 3878 and 4656 DEGs for CA, and 3135 and 4316 DEGs for YH72 at CT4 and CT8, respectively, with 731 and 1130 common DEGs between CT4 and CT8 (Figure 4G, H). Apparently, more DEGs were induced by the low temperatures in the high‐LTG group than in the low‐LTG group. Detailed comparison of the DEGs between the low and high‐LTG groups revealed that 5390 DEGs were simultaneously detected in both groups in CT4 and 7149 DEGs in CT8, with 5866 and 6631 DEGs specifically identified in CT4 and CT8, respectively (Figure 4I, J). Also, the number of commonly detected DEGs in subspecies *Xian* and *Geng* was 5758 in CT4 and 7564 in CT8, while 5498 and 6216 DEGs were specifically identified in CT4 and CT8, respectively (Figure 4K, L).

**Figure 4:**
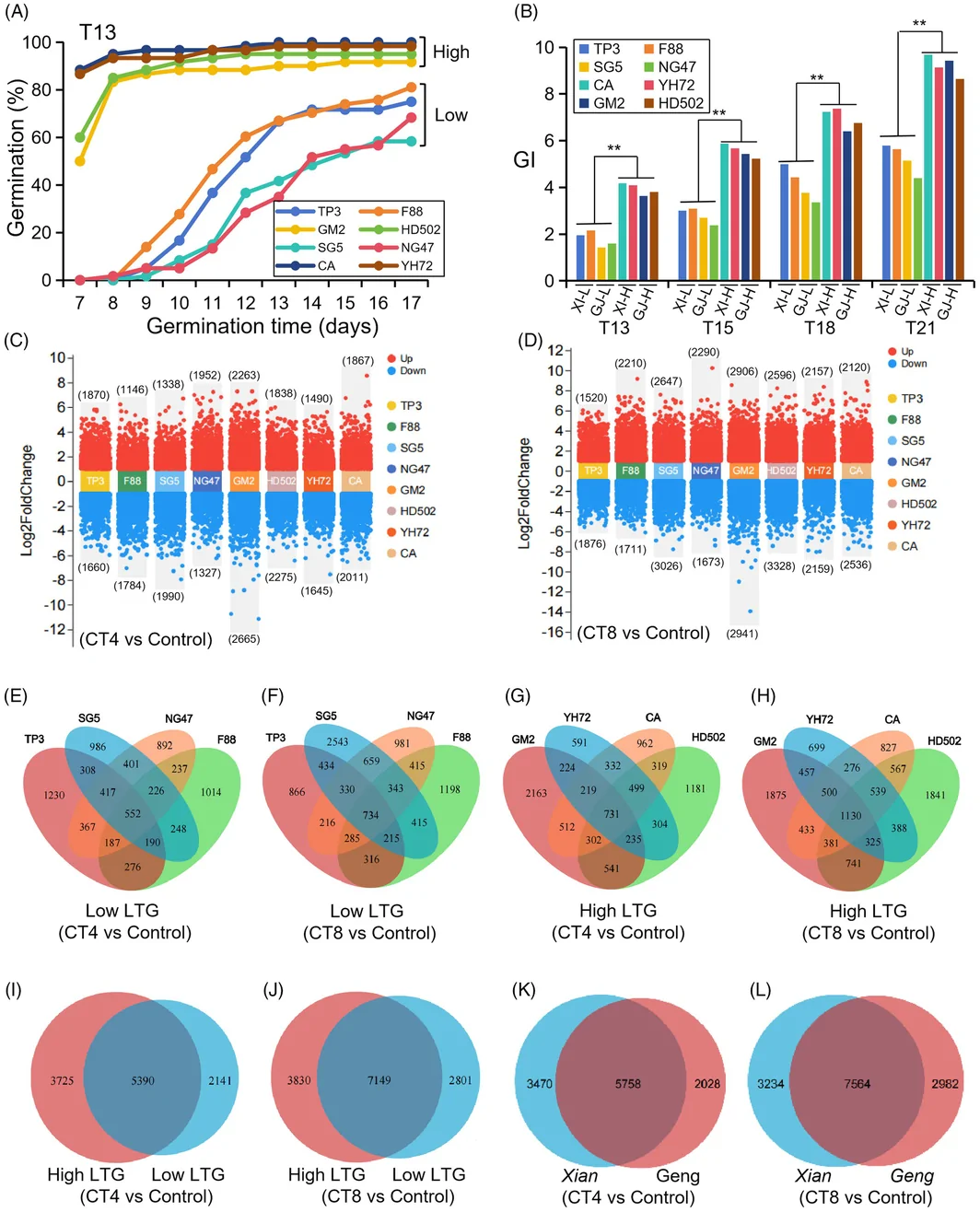
The transcriptome analysis of eight rice accessions differing in their LTG. (A, B) the LTG performance of the eight rice accessions. (C–D) Volcano plot of differentially expressed genes (DEGs) between the check and after 4 h (C) and 8 h (D) low‐temperature treatment. Red dots denote upregulated genes; Blue dots indicate downregulated genes. (E–J) Comparison of DEGs numbers in low‐LTG and high‐LTG accessions. (K–L) Comparison of DEGs numbers in *Xian* and *Geng* subspecies.

### Elite alleles of the candidate genes contributing to high LTG

For the 11 important QTL consistently detected across all four temperatures by SNP–GWAS, we identified candidate genes by integrating three lines of evidence: (1) positional genes within each QTL interval after removing transposons and non‐expressed genes; (2) DEGs commonly induced at both CT4 and CT8 from RNA‐seq; and (3) haplotypes constructed from SNPs in promoter and coding regions that showed significant phenotypic differences among major haplotypes (Duncan test, *P* < 0.05). This integrated approach yielded 25 candidate genes and 43 favorable haplotypes, defined as haplotypes with significant positive effects on LTG (Table S10). For *qLT1k*, the strongest candidate *OsABI5* (Os01g0859300), encoding a bZIP transcription factor, exhibited four major haplotypes with significant phenotypic differentiation: three favorable haplotypes (Hap1, Hap3, Hap4) associated with high LTG were enriched in low‐latitude *Xian* accessions, while Hap2 (low‐LTG) predominated in high‐latitude *Geng* accessions; *OsABI5* was significantly upregulated by cold treatment (CT) in high‐LTG accessions but unresponsive in low‐LTG lines. For *qLT2k* and *qLT2n*, we prioritized three and two candidate genes, respectively, with favorable haplotypes again enriched in low‐latitude *Xian* accessions; four of these five genes were CT‐induced in high‐LTG but not low‐LTG accessions, whereas *OsPP2C51* (Os05g0572700, *qLT5h*), encoding a clade A phosphatase 2C, was induced by CT in both groups, with its high‐LTG haplotypes (Hap1–3) enriched in low‐latitude *Xian* accessions. For *qLT7j*, three candidate genes were prioritized, with five high‐LTG favorable haplotypes enriched in high‐latitude *Xian* accessions and all genes induced by CT in both LTG groups. At *qLT8a*, the single candidate Os08g0113300 (encoding a STIP1 homology and U box‐containing protein 1) showed Hap2–3 as predominant high‐LTG haplotypes in *Xian* accessions and was downregulated by CT in high‐LTG *Xian* and *Geng* accessions but induced only in low‐LTG Geng accessions. For *qLT9e*, three candidate genes displayed two major haplotypes each with significant phenotypic differences; their favorable high‐LTG haplotypes were concentrated in low‐latitude *Xian* accessions, with distinct CT responses: Os09g0394700 was downregulated in both groups, Os09g0395300 induced in high‐LTG *Geng* accessions only, and Os09g0395600 uninduced in high‐LTG but repressed in low‐LTG accessions. For *qLT10g*, the favorable haplotype Os10g0407200^Hap3^ was enriched in low‐latitude *Xian* accessions, with Os09g0395600 downregulated in most high‐LTG and one low‐LTG line; *qLT10i* carried six glutathione S‐transferase family candidate genes, with seven favorable haplotypes enriched in low‐latitude *Xian* accessions and all genes induced by CT in both LTG groups. Finally, for *qLT11b*, which represents one of the two key QTL (*qLT11b* and *qLT11d*) on chromosome 11, three candidate genes were prioritized, all induced by CT in high‐ and low‐LTG accessions, with their favorable haplotypes predominantly found in low‐latitude *Xian* accessions.

For the five important loci independently detected by SV–GWAS (*qLTv4k*, *qLTv5i*, *qLTv7g*, *qLTv9g*, and *qLTv11l*), we further performed haplotype composition and LTG phenotypic comparison analyses. Since SVs are mostly large‐fragment variations, if an SV was located within a gene region, that gene was prioritized as a candidate gene. The candidate gene for *qLTv4k*, Os04g0647425, encodes an unknown function protein containing a DUF247 plant domain; its Hap1 showed significantly higher values than Hap2 for LTG parameters at T13, T15, T18, and T21, and significantly lower values than Hap2 for GT50. The candidate gene for *qLTv5i*, Os05g0565900, encodes a protein containing a DNA‐binding WRKY domain; the candidate gene for *qLTv7g*, Os07g0593000, encodes a C2H2 zinc‐finger transcription factor; and the candidate gene for *qLTv9g*, Os09g0441900, encodes a functional protein containing a PEBP‐like domain. For these three loci, Hap1 likewise showed significantly higher germination rate parameters and significantly lower GT50 than Hap2, representing favorable haplotypes for high LTG. The candidate gene for *qLTv11l*, Os11g0686250, encodes a protein containing a DNA‐binding WRKY domain; its Hap1 also showed an advantage in germination rate parameters, but the GT50 difference was relatively small. Among them, Hap1 was identified as the favorable haplotype for high LTG at all five loci. Cold‐treatment RNA‐seq further showed that *qLTv4k* was mostly downregulated in Hap1 high‐LTG materials; *qLTv5i* and *qLTv7g* were cold‐induced in Hap1 high‐LTG materials; and although *qLTv9g* and *qLTv11l* showed clear phenotypic differentiation, their transcriptional responses were relatively weak.

To test whether expression responses to cold differed among haplotypes, we grouped CT4 and CT8 expression changes of the 30 candidate genes from eight RNA‐seq accessions by haplotype, excluding rare haplotypes. Among 358 valid observations (120 favorable vs. 238 unfavorable), mean expression changes were 0.351 and 0.152, respectively, with no significant difference overall (*P* = 0.187) or at CT4/CT8 (*P* = 0.704/0.168). Only three genes, Os05g0572700, Os10g0530400, and Os11g0156200, showed significantly higher expression for favorable haplotypes (*P* < 0.05). The remaining genes showed no differences between haplotype groups.

### Genes functional validation and natural variation analysis of the *qLT11d*

*qLT11d* was detected in six LTG‐related traits under four temperature treatments and was the locus with the largest effect among multiple LTG traits (Table S6). Combined with the candidate interval of *qLT11d* through transcriptional expression analysis and excluding genes with no LTG phenotypic differences among haplotypes in the candidate region, Os11g0438000 (https://rapdb.dna.affrc.go.jp/) was finally identified as the candidate gene of *qLT11d* (Figure 5A–C). This gene encodes a putative expressed tropinone reductase (https://www.ricedata.cn/). Two homozygous mutants (designated KO1 and KO2) were generated via gene editing. KO1 inserted “A” base at the first editing site and deleted two bases at the second editing site. KO2 inserted one “G” base at the first editing site and “A” base at the second editing site, both resulting in premature translation termination (Figure 5A, D). Germination curve analysis showed that under cold treatment, the seed germination rate and seedling emergence rate of *qlt11d* knockout lines were significantly lower than those of the WT Nipponbare (NIP) (Figure 5E), demonstrating that *qLT11d* is a positive regulator of LTG in rice. Specifically, GT50 was significantly prolonged in both KO1 and KO2 across all temperatures, with the greatest delays at 13°C (WT: 298.5 h; KO1: 338.9 h; KO2: 390.5 h) and 15°C (WT: 232.7 h; KO1: 261.2 h; KO2: 290.1 h) (Table S9). GT50 was also significantly prolonged in both knockout lines at 18°C and 21°C (Table S9). Consistently, MGT was also significantly increased in both knockout lines relative to NIP across all temperatures (Table S9). Moreover, at the final observation points, root length and shoot length of KO1 and KO2 were significantly (*P* < 0.05) lower than those of WT (Table S9). Haplotype analysis results revealed that Hap1 of *qLT11d* was highly enriched in *Geng* accessions from high‐latitude regions (accounting for 96.0%) and associated with the lowest LTG phenotype (Figure 5F, G). In contrast, only 1.0% of accessions in the *Xian* subgroup carried Hap1 (Figure 5H, I). This haplotype distribution pattern underscores the adaptive divergence of LTG between *Xian* and *Geng* subspecies and identifies *qLT11d* as a promising locus for targeted improvement of low‐temperature germination (LTG) in rice breeding.

**Figure 5:**
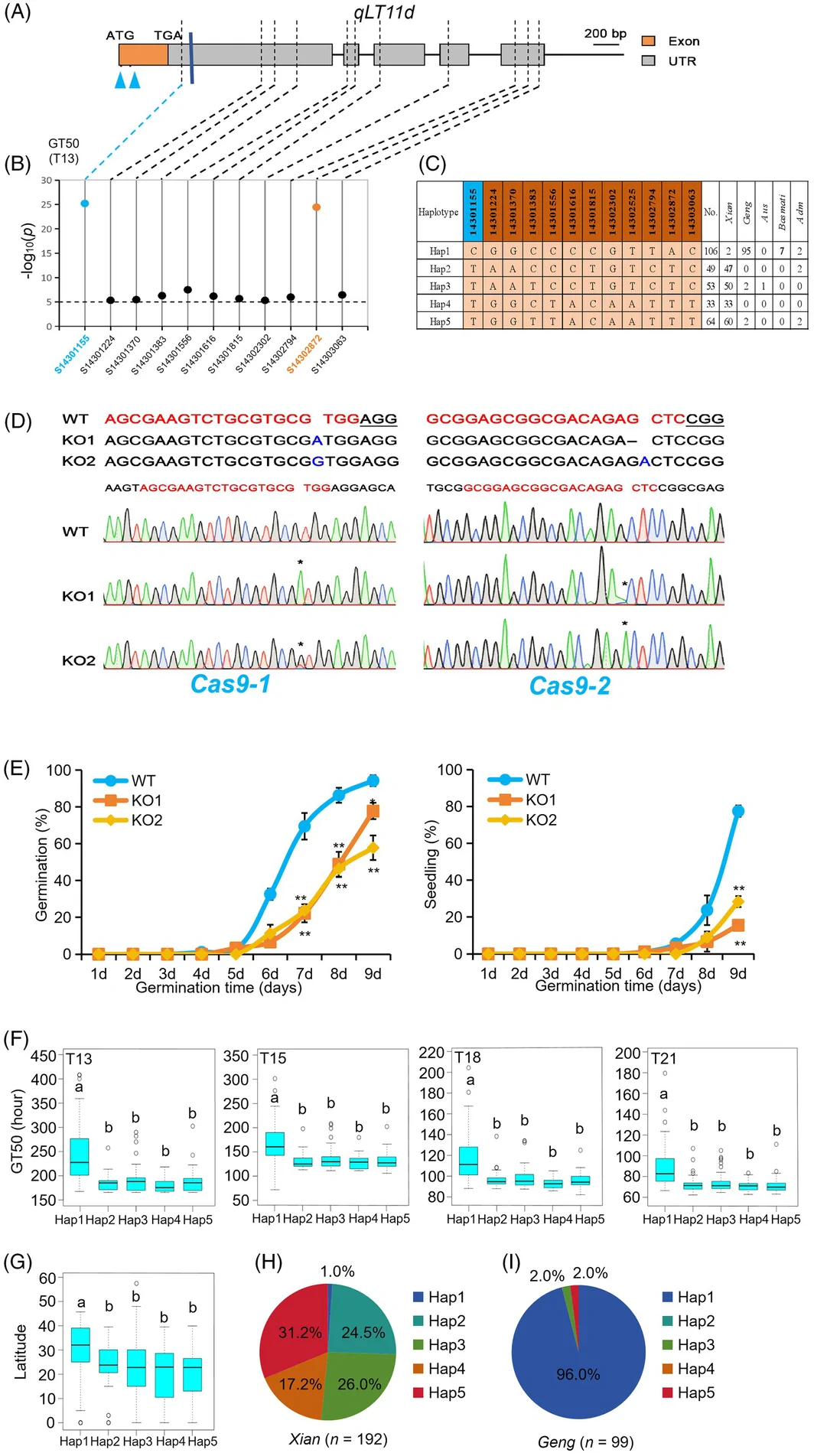
Functional validation of the major‐effect gene *qLT11d* in regulating low‐temperature germination (LTG) in rice. (A) Schematic diagram of the *qLT11d* gene structure. The two blue triangles denote the Cas9‐1 and Cas9‐2 target sites. (B) Gene‐based GWAS for LTG. Horizontal dashed black line corresponds to a reference threshold (−log10(*P*) >5.0). The orange and a blue (peak SNP) dots indicate major SNPs that are significantly associated with LTG. (C) Haplotype analysis of *qLT11d* based on the major SNPs in the gene coding region. The accessions carrying any one of the missing genotype were ignored. (D) Schematic diagram of the gene‐editing target sites for *qLT11d*. (E) Dynamic germination and seedling emergence phenotypes of dormant seeds from the *qLT11d* knockout transgenic lines and wild‐type (WT) under low‐temperature conditions. (F) Phenotypic differences in GT50 among different haplotypes of *qLT11d*. Different lowercase letters (a, b) indicate extremely significant differences based on Duncan's test (*P* < 0.01). (G) Geographical distribution differences of germplasms harboring different haplotypes of *qLT11d*. (H, I) Proportions of different haplotypes of *qLT11d* in the *Xian* (H) and *Geng* (I) subspecies.

### Presence–absence variations (PAVs) of the LTG candidate genes

To determine how gene PAVs of the 25 candidate genes (identified via SNP–GWAS) contributed to LTG, we performed gene PAV analysis in 539 rice accessions (six *Wild*, 359 *Xian*, 103 *Geng*, 48 *Aus*, and 23 *Bas* accessions) (downloaded from (Wang et al., 2018; Zhang et al., 2022)). Of the 25 candidate genes, 10 were core genes present in all 539 accessions, eight were candidate core genes present in >98% of the accessions, and seven (28.0%) were distributed genes (Figure 6A, B). The 25 gene PAV data could clearly divide the 539 rice accessions into four types. The six *Wild* rice accessions were clustered in type II, *Xian* and *Aus* accessions were mainly distributed in types I, II, and III, the *Bas* accessions were highly enriched in types II and III, while most *Geng* accessions were clustered in type IV. The favorable alleles for high LTG at the candidate genes showed a similar pattern (Table S10). These results indicated that gene PAVs are correlated with the *Xian–Geng* subspecific differentiation, although the causal direction remains to be determined. For each distributed gene, Os02g0727100 was present in 173 of the 539 accessions. Os02g0727100 was present in all *Geng* accessions and most of *Wild* and *Bas* accessions. Os07g0601100 was present in only 113 accessions, including all *Wild* accessions. The remaining five genes (Os09g0394700, Os09g0395300, Os10g0527800, Os10g0528400, and Os11g0154900) were present in >70% of the accessions. Almost all *Geng* accessions have Os10g0527800 and Os10g0528400, which were absent in all *Wild* accessions except that Os10g0528400 was present in one wild accession (Figure 6C, D). When GWAS using 84 additional accessions to test associations of the gene PAVs with the LTG phenotype (T15), the presence of three candidate genes (Os02g0727100, Os010g527800, and Os10g0528400) was significantly (*P* < 0.005) associated with increased LTG (Figure 6E–P). These results indicated that gene PAVs were an important contributor to the phenotypic variation of LTG in rice populations.

**Figure 6:**
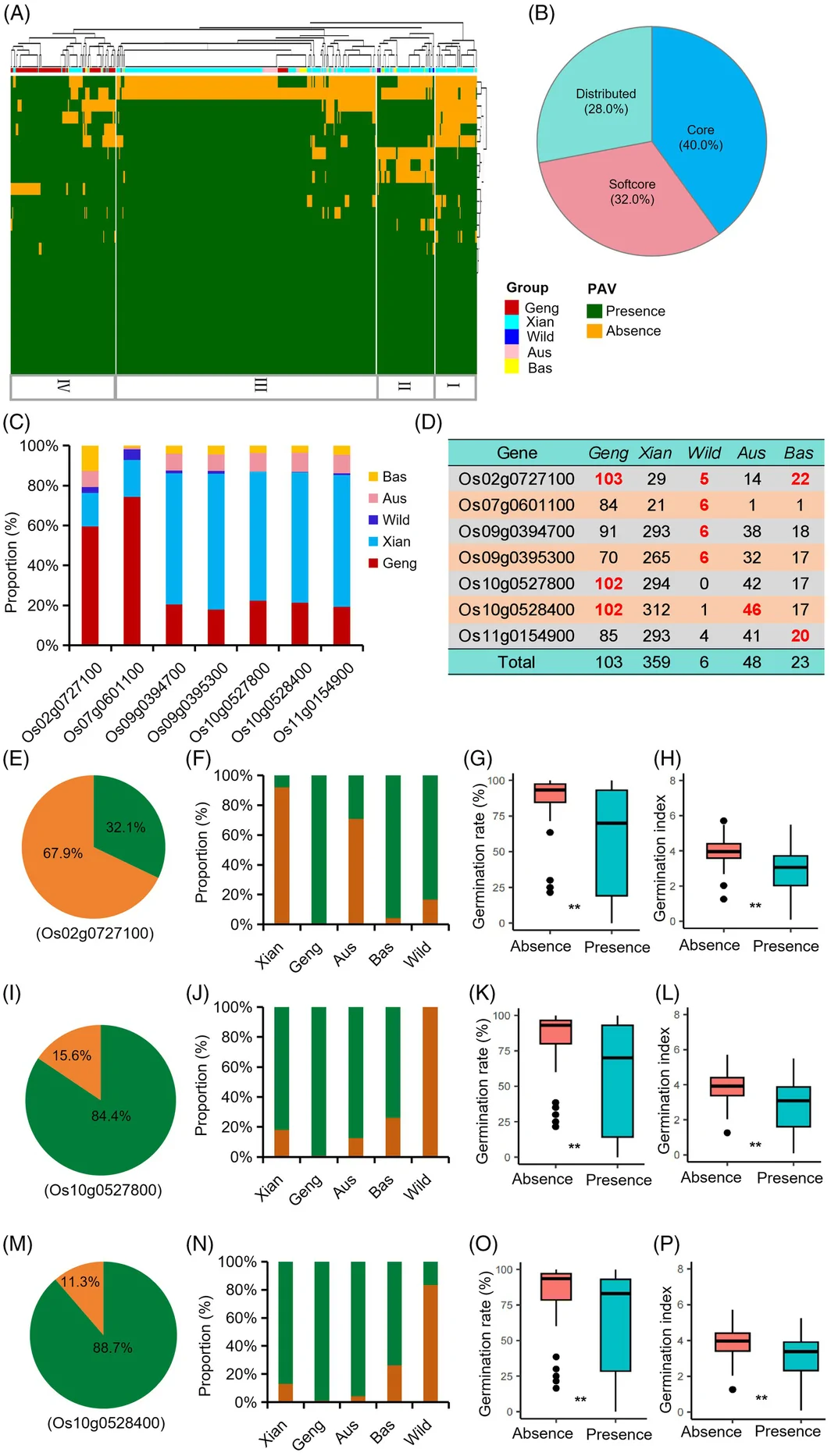
Candidate gene presence–absence variations (PAVs) analysis in 539 rice accessions. (A) The heatmap of 25 candidate gene PAVs in 539 rice accessions. The gene PAV information of 539 rice accessions including six wild, 359 *Xian*, 103 *Geng*, 48 *Au*s, and 23 *Bas* accessions was obtained from 453 rice accessions sequenced with next‐generation sequencing (NGS) (Zhang et al., 2022) and 110 rice accessions sequenced with third‐generation sequencing (ignoring 24 admix accessions) (Zhang et al., 2022). (B) The proportion of the 25 candidate genes. Core gene (present in all 539 accessions); softcore/candidate core gene (present in more than 90% of accessions); and distributed gene (present in less than 90% but more than one of the accessions). (C–D) The distribution of seven distributed genes in the five subspecies. The red text represents the gene highly (more than 90% accessions) presented in the corresponding subspecies. (E–P) Three examples of distributed gene PAVs (Os02g0727100, Os010g0527800, and Os10g0528400) significantly associated with LTG phenotype. (F, J, N) The proportions of the rice accessions carrying the corresponding gene PAVs in five subspecies. The green and orange represent gene presence and absence, respectively. Wilcoxon rank‐sum test was used to detect gene PAV‐phenotype associations. “**” denote *P*‐value <0.001.

### Genetic architecture of LTG

As briefly revealed in above sections, the geographic locations, particularly the latitudes the 403 rice accessions locate were negatively correlated with their assayed LTG parameters across all four temperatures. Pearson correlation analysis showed that most LTG parameters for the whole population were significantly correlated with latitude of origin across all four temperatures (Figure 7A). Latitude correlated negatively with germination‐related parameters and positively with time‐related parameters such as GT50 and GMGT, with absolute *r* values ranging from 0.27 to 0.45 across temperatures. We further examined whether the observed latitude–LTG correlations were driven by population structure by performing subpopulation‐specific analyses in *Xian* (*n* = 259) and *Geng* (*n* = 121). Across all temperatures and LTG‐related parameters, latitude was significantly correlated with each trait in both subpopulations (all *P* < 0.05; Table S11). In the *Xian* subpopulation, the absolute PCCs ranged from 0.16 to 0.85, with a mean of 0.39; in the *Geng* subpopulation, they ranged from 0.20 to 0.83, with a mean of 0.46. Both mean values were significantly higher than that of the whole population (0.24; *P* < 0.01). These results indicate that the association between latitude and LTG is not merely a consequence of subspecies differentiation, but remains robust within each subpopulation. Particularly, rice accessions from areas of low latitudes showed significantly (*P* < 0.01) higher LTG than the accessions of the higher latitude areas (Figure 7B). This was reflected by their significantly higher frequencies of the favorable alleles for high‐LTG at the 25 candidate genes (identified above and summarized in Table S10) than those from areas of low latitudes (Figure 7C, D). In fact, *Xian* accessions of low‐latitude areas carry high frequencies of the alleles for high LTG, whereas most *Geng* accessions from high‐latitude regions tended to have low‐LTG and high frequencies of low‐LTG alleles at the candidate genes (Figure 7E). This was better illustrated by the genotypic comparison at the candidate genes between seven accessions of extremely high LTG and seven accessions of extremely low LTG (Figure 7F), which showed that the high‐LTG accessions possess the high‐LTG alleles at most of the candidate genes, while the opposite was true for the low‐LTG accessions. This result suggested that the pyramiding of more high‐LTG alleles at the candidate genes could be an effective strategy for developing rice varieties with high LTG.

**Figure 7:**
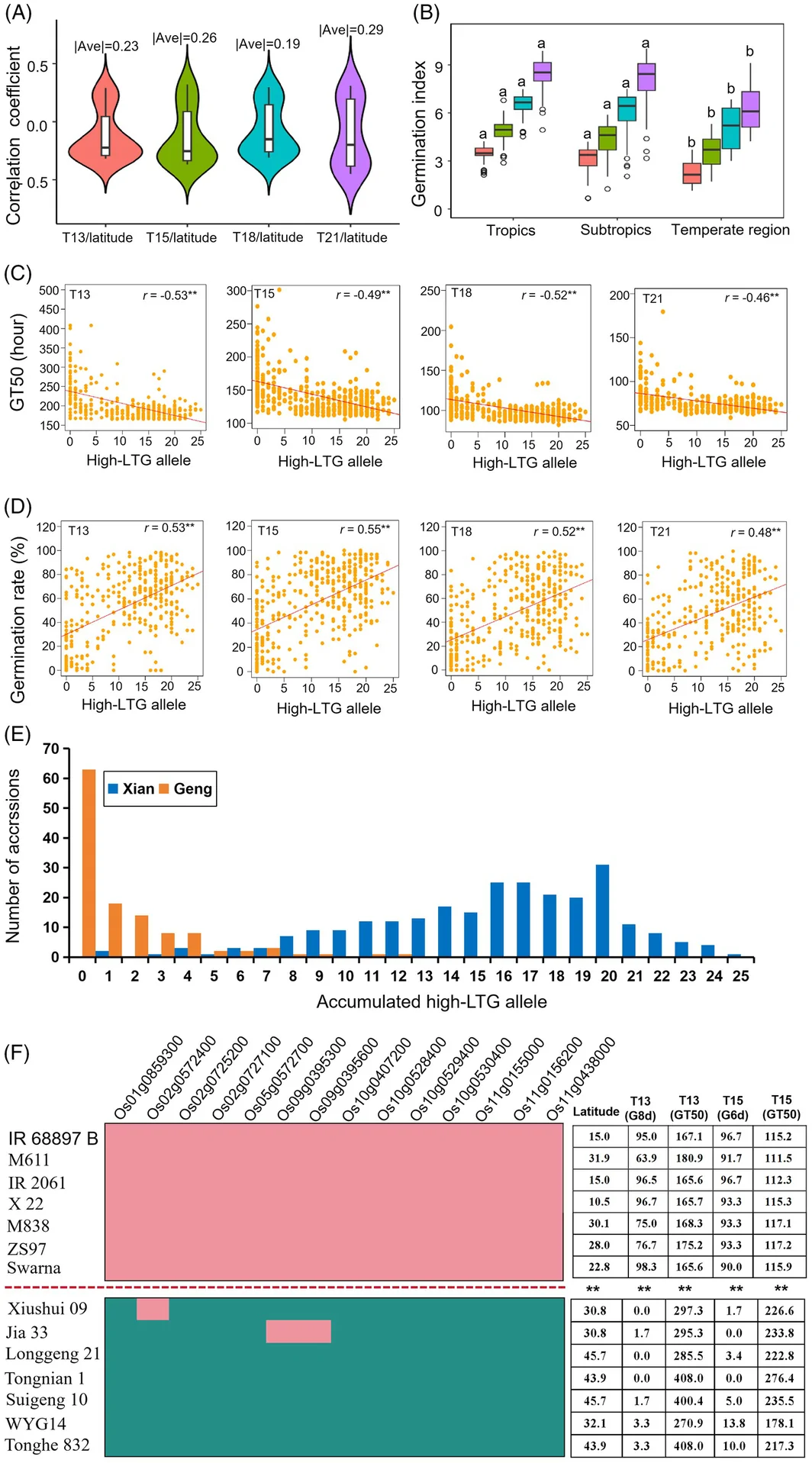
Genetic architecture of low‐temperature germination (LTG). (A) Average correlation coefficients between the latitude distribution and LTG in four temperatures. (B) Germination index performance of the corresponding rice accessions collected from tropics, subtropics and temperate regions. The red, green, blue, and purple boxes represent the Germination Index (GI) of the accessions in T13, T15, T18, and T21, respectively. Different letters denote significant differences in LTG among accessions from different latitudinal origins under the same temperature condition (*P* < 0.05). (C, D) Plot of the LTG level against the accumulation of high‐LTG alleles. The *x*‐axis indicates the number of accumulated high‐LTG alleles from all candidate genes in the rice accessions; the *y*‐axis shows the germination parameter (C) and GT50 parameter (D). (E) Number of the accumulated high‐LTG alleles in *Xian* and *Geng* subspecies. (F) High‐ and low‐LTG allele numbers in seven high‐LTG accessions and seven low‐LTG accessions at 14 candidate genes. The red pane indicates favorable allele. The green pane indicates unfavorable allele. The dotted line separates the high‐LTG and low‐LTG accessions.

### Inter‐ and intra‐subspecies genetic differentiation of LTG

To further reveal the genetic basis of LTG in rice, we investigated the genome‐wide inter‐ and intra‐subspecific differentiation in LTG by population differentiation statistics (F_ST_). First, we selected 60 *Xian* accessions (*Xian*‐H) with the highest LTG and 49 *Geng* accessions (*Geng*‐H) with the lowest LTG across the four temperatures, and calculated the F_ST_ statistics genome‐wide between these two groups (Figure 8A, B). This resulted in the identification of 61 highly divergent genomic regions (Figure 8C; Table S13). When the 61 regions were compared with previously identified LTG QTL, 29 common regions were identified and named as DR_LGs (Figure 8E). The 29 DR_LGs covered 28.2 Mb of the rice genome (Table S13), 15 of which were exclusively detected by the SNP‐based GWAS, four were specifically detected by the SV‐based GWAS, six were commonly detected by both, and eight were reported previously. The Tajima's *D* and nucleotide diversity estimates at these DR_LGs indicated that eight of them were apparently under selection, at which population *Xian* showed significantly higher average genetic diversity (π = 0.00072) than population *Geng* (π = 0.00040) with mean π_*Xian*_/π_*Geng*_ = 1.49 (Table S13).

**Figure 8:**
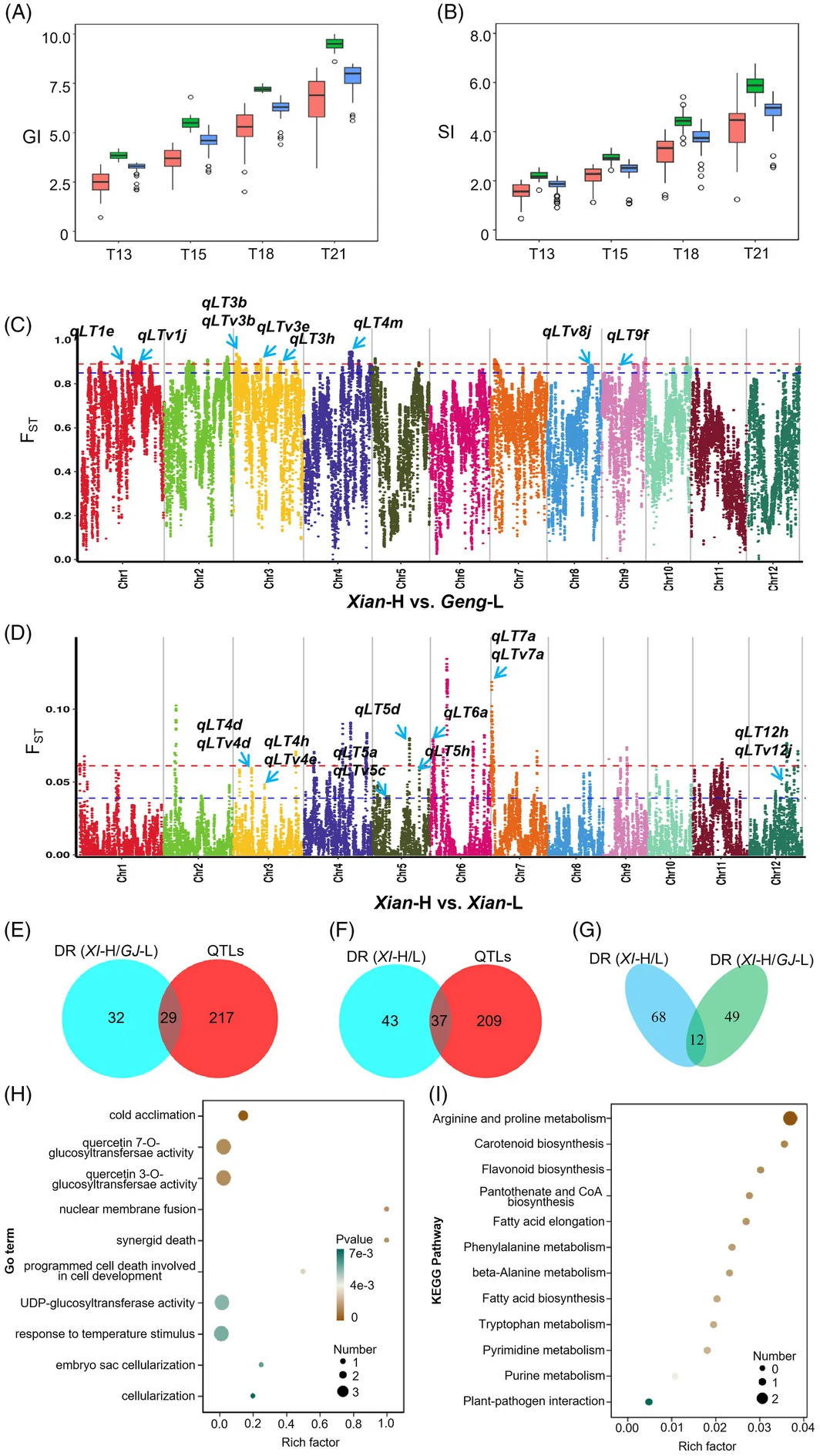
Genomic differentiation of LTG. (A, B) GI and SI of the accessions with the highest LTG (*n* = 60) and the accessions with the lowest LTG (*n* = 58) from *Xian*, and the accessions with the lowest LTG from *Geng* (*n* = 49). (C) Genomic differentiation of the *Xian* and *Geng* subspecies. *Xian*‐H and *Geng*‐L represent the accessions with the highest LTG from *Xian* and the accessions with the lowest LTG from *Geng*. (D) Genomic differentiation of the *Xian* subspecies. *Xian*‐L represents the accessions with the lowest LTG from *Xian*. The blue arrow and text denote the divergent regions (DRs) overlapping with the QTLs/candidate genes identified by GWAS in this study. Horizontal red and blue dashed lines correspond to the top 1 and 5% thresholds, respectively. (E, F) Comparison of the DRs detected *Xian–Geng* (E) and intra‐*Xian* (F) with the QTL identified in this study (B–D) and QTLs and cloned genes in previous studies. (G) Comparison of the DRs detected *Xian–Geng* and intra‐*Xian* for LTG. (H, I) KEGG and GO analysis of the DEGs that were located in DRs.

To understand the genetic basis of LTG within population *Xian*, we compared the genome‐wide differentiation between the 60 high‐LTG (*Xian*‐H) accessions and 58 low‐LTG (*Xian*‐L) accessions (Figure 8A, B), resulting in the identification of 80 highly divergent genomic regions between *Xian*‐H and *Xian*‐L (Figure 8D; Table S14). When these regions were compared with the identified LTG QTL, 37 DR‐LGs overlapped with 28 identified QTL, including 17 LTG QTL reported previously (Figure 8F; Table S15). The 37 DR_LGs covered 7.3% (27.3 Mb) of the NIP genome. The 28 overlapping QTL included 13 QTL detected exclusively by SNP‐based GWAS, four specifically detected by SV‐based GWAS, 11 common ones detected by both, plus eight QTL commonly detected in this and previous studies (Table S15).

A comparison of the DRs identified in the *Xian*‐H/L and *Xian‐*H*/Geng‐*L revealed 12 overlapping genomic regions (Figure 8G). Among them, six identified LTG QTL located in four of the DRs. For example, *qLT3b/qLTv3b/qLTv3c* showed major effects on the LTG parameters identified through SNP‐ and SV‐based GWAS. Two large‐effect QTL, *qLT6h* and *qLTv9g*, were identified by SNP‐ and SV‐based GWAS. In addition, the overlapping DRs also contained four previously reported LTG QTL (Table S16). These results indicated that some genomic regions were selected in both inter‐ and intra‐subspecies.

### The DEGs overlapping with DR regions

To obtain additional evidence to support the identified candidate genes in the DR_LTG regions, we sorted all DEGs commonly identified at CT4 and CT8 from each assayed accession into three categories: (1) DEGs that were uniquely expressed in the two sensitive and two tolerance *Xian* accessions to identify DEGs in the DRs differentiating *Xian*‐H/*Xian*‐L; (2) DEGs that were specifically expressed in the four *Xian* and four *Geng* accessions to detect DEGs in the DRs that differentiated *Xian*‐H/*Geng*‐L; and (3) their intersections to identify DEGs in the 12 common DRs from *Xian*‐H/*Xian*‐L and *Xian*‐H/*Geng*‐L. We focused only on the DRs that were overlapped with the identified QTL or previously reported QTL. Finally, 62 DEGs were identified from *Xian*‐H/*Xian*‐L, and 20 DEGs were identified from *Xian*‐H/*Geng*‐L (Tables S13, S15 and S16). The enriched GO terms revealed that these DEGs were mainly related to response to temperature stimulus, cold acclimation, UDP‐glucosyltransferase activity, quercetin 7‐O‐glucosyltransferase activity, quercetin 3‐O‐glucosyltransferase activity, nuclear membrane fusion, synergid death, programmed cell death involved in cell development (Figure 8H). The KEGG enrichment analysis showed that the important pathways involved were arginine and proline metabolism, carotenoid biosynthesis, flavonoid biosynthesis, pantothenate and CoA biosynthesis, and fatty acid elongation (Figure 8I).

### Machine learning‐based genomic selection (GS) system for LTG prediction

Based on the SNP and SV datasets of 403 rice accessions, the fusion model MFMGP software constructed with multiple machine learning approaches (https://mfmgp.bioaibp.com/#/Breeding/MFMGP/) was applied to predict the GT50 index under four temperature gradients (T13, T15, T18, T21). GT50 was selected as the target phenotype among the 39 LTG parameters because it is a widely used, interpretable indicator of germination speed and uniformity, key traits for direct seeding, and strongly correlates with most other germination and seedling parameters (average |*r*| > 0.75, *P* < 0.01), thereby capturing the bulk of LTG variation, and was stably detected by GWAS across all four temperatures and multiple panels, confirming its robust genetic basis. The results showed that the prediction accuracies (Pearson correlation coefficient, PCC) of the SNP dataset at T13, T15, T18, and T21 were all above 0.6, with the values of 0.69, 0.63, 0.69, and 0.62, respectively; the corresponding prediction accuracies of the SV dataset at the above temperatures were 0.71, 0.63, 0.70, and 0.67, respectively (Figure 9A). Among them, the prediction accuracy of the SV dataset for the GT50 index at T13 and T21 was significantly higher than that of the SNP dataset (*P* < 0.05), while no significant difference was observed in the prediction accuracy between the two datasets at T15 and T18. This indicated that in addition to SNPs, SVs also exhibited good performance in GS. A comparison of the prediction accuracies of the two datasets across different temperatures revealed that the prediction accuracies at T13 and T18 were similar, and both were significantly higher than those at T15 and T21 (Figure 9A). The results of prediction stability analysis demonstrated that the model stability was improved with the increase in temperature, along with a decrease in the RMSE (Figure 9B). Therefore, temperature had a notable effect on both the prediction accuracy and model stability of the GT50 index associated with LTG in rice. To evaluate model generalizability, we validated the model using 84 external accessions using the SV dataset (Table S17). The predicted values were significantly correlated with the observed values (*r* = 0.52, *P* < 0.01), indicating that the model has a certain cross‐population prediction ability. Within‐subpopulation validation yielded a prediction accuracy of 0.58 (*P* < 0.01) for *Xian* and 0.46 (*P* < 0.01) for *Geng*. In cross‐subpopulation validation, the accuracy was *r* = 0.20 when training on *Xian* and predicting *Geng*, and *r* = 0.19 when training on *Geng* and predicting *Xian* within the training set. For external accessions, cross‐subpopulation prediction accuracies ranged from *r* = 0.13 to 0.18, none reaching statistical significance (*P* > 0.05), suggesting that genetic divergence between *Xian* and *Geng* limits the cross‐subpopulation application of the model.

**Figure 9:**
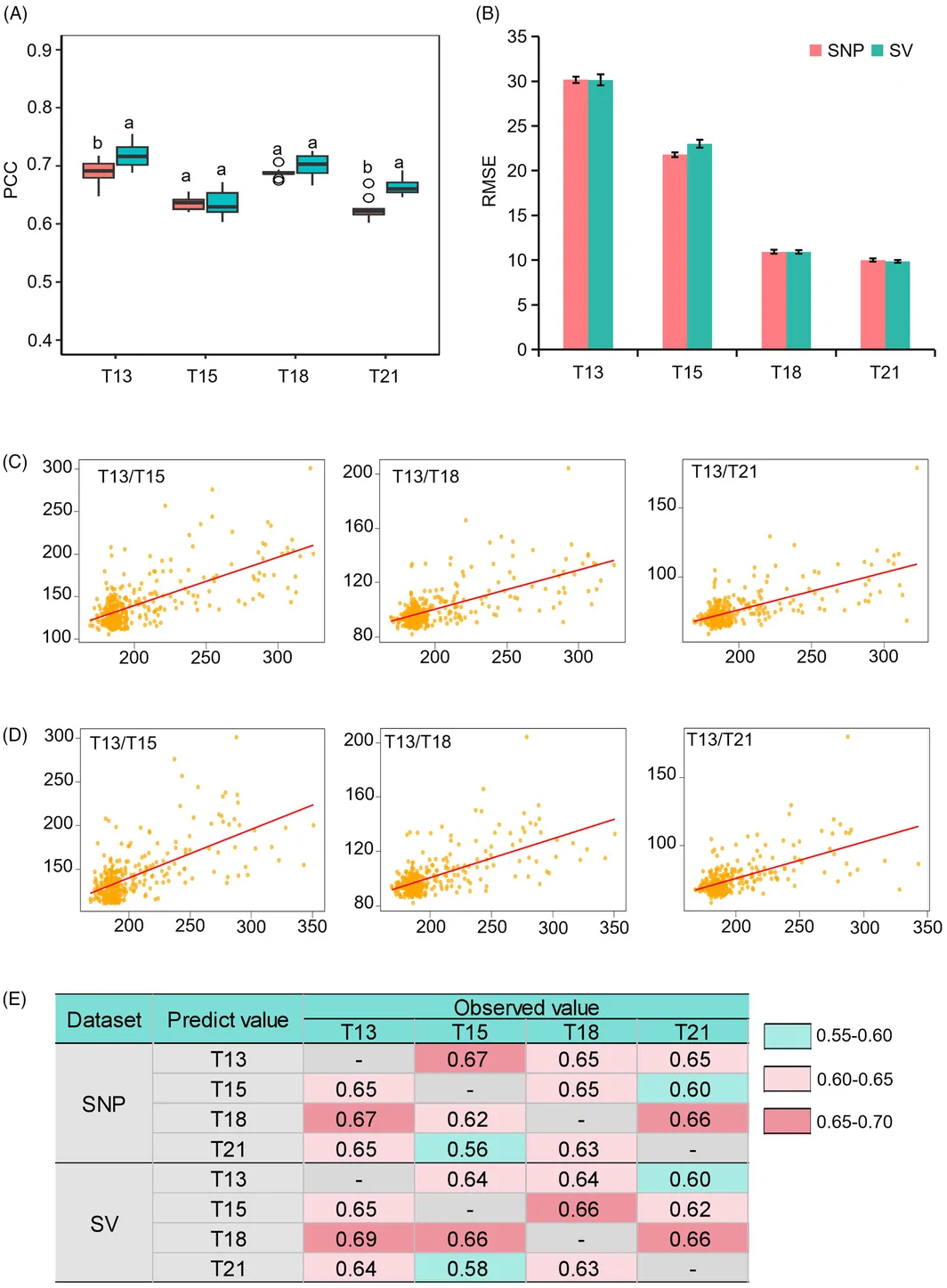
Construction of a genomic selection model for LTG traits based on the MFMGP fusion machine learning model using SNP and SV datasets. (A) Prediction accuracy of LTG traits (GT50) at four temperatures in 403 rice accessions using the MFMGP model. Red and green represent the prediction accuracy of SNP and SV datasets, respectively. (B) Performance of prediction stability for LTG traits at four temperatures, as evaluated by RMSE. (C, D) Correlation analysis between predicted LTG values at T13 and observed values at T15, T18, and T21 based on SNP (C) and SV (D) datasets. (E) Correlation analysis between predicted values under a single temperature and observed values under the other three temperature treatments.

Furthermore, we evaluated the cross‐temperature prediction ability of the model by calculating the correlation coefficients between predicted values under a single temperature and observed values under the other three temperatures. The results showed that, regardless of using the SNP or SV dataset, the correlation coefficients between predicted LTG (GT50) values at each temperature and observed values at the other three temperatures were all above 0.55 (*P* < 0.01) (Figure 9C–E). For instance, when the training set was built with the SNP dataset, the correlation coefficients between predicted GT50 values at T13 and observed values at T15, T18, and T21 were 0.67, 0.65, and 0.65, respectively (Figure 9C); when the training set was constructed with the SV dataset, the corresponding correlation coefficients were 0.64, 0.64, and 0.60, respectively (*P* < 0.01) (Figure 9D). Notably, the average correlation coefficients between predicted values at T18 based on the SNP and SV datasets and observed values at the other three temperatures were as high as 0.65 and 0.67, respectively (Figure 9E), indicating that the model showed superior cross‐temperature prediction performance for LTG at other temperatures when T18 was used as the treatment condition.

## DISCUSSION

### SVs are extremely useful markers for discovering QTL/genes affecting complex traits

LTG is not only an agronomically important but also a crucial adaptive trait, which makes improved germination ability an imperative in rice breeding. Rapid and uniform seed germination and seedling rate under a low‐temperature condition is a desirable characteristic of rice production practice. LTG is a typical complex quantitative trait controlled by multiple QTL/genes and is greatly affected by environments. However, only few of QTL have been cloned in rice, and thus molecular mechanism underling LTG is still largely unclear. Therefore, identification and characterization of LTG QTL and their candidate genes are essential for understanding the genetic basis of LTG in plants. Using 403 rice accessions collected from 27 countries, we were able to identify 200 QTL and large numbers of candidate genes associated with multiple LTG parameters. In addition, we also identified 58.9% regions associated with LTG that have not been reported before. This number was far greater than the LTG QTL reported previously, which was at least partially attributed to our use of the SV‐based GWAS as most QTL identified by the SV‐based GWAS were different from those identified by SNP‐based GWAS. A similar result was reported in tomato where only 5.2% of the QTL identified by SV‐ and SNP‐based GWAS are common ones (Li, He, et al., 2023). Nevertheless, although SV‐based GWAS identified additional QTL, these loci were not systematically characterized previously. Given that these findings remain correlative, we view SV‐based GWAS primarily as a hypothesis‐generating tool that complements SNP‐based discovery, rather than a means of definitive gene identification. Functional validation (e.g., gene editing, NILs) is required to establish causal links between specific SVs and LTG. Thus, while integration of SNPs and SVs can serve as a potential complement that enriches the candidate pool, its power depends on subsequent validation efforts. These results indicate that the SV‐based GWAS can serve as a potential complement that enriches the candidate pool, but its power depends on subsequent validation efforts. Thus, integration of SNPs and SVs is an effective strategy to elucidate the genetic basis of complex traits, including LTG. Notably, haplotype and cold‐responsive expression data jointly revealed that transcriptional differences between favorable and non‐favorable haplotypes were not significant for most candidate genes, including *qLT11d*. This suggests that natural variation in rice LTG is not primarily attributable to differential transcript abundance but may involve at least two distinct mechanisms: expression‐driven regulation and functional variation in coding or regulatory regions that affects protein activity independently of transcriptional changes. For several major candidates, particularly *qLT11d*, the evidence leans toward the latter. Thus, prioritizing candidate genes solely by expression differences in haplotype‐based studies may introduce bias, and future work should integrate coding‐sequence variant effect prediction and protein functional assays to clarify causal mechanisms.

Several interesting characteristics of SVs were noted. First, the homozygous genotype had a lower frequency than the NSV genotype at most SVs. Second, panel *Geng* had a much higher (3.3‐fold) average SV frequency than panel *Xian*. Third, panels *Xian* or *Geng* each contain a large number of specific SVs, and the majority (average 55%) of these subspecies‐specific SVs had low frequencies within the subspecies. Because of these properties of SVs, one may have two expectations on QTL identified by SVs. First, using SVs as markers has generally a low power to detect QTL in random populations as compared with SNPs, as we observed in this study. Second, most SV‐detected QTL tend to occur within target QTL genes because of their large phenotypic effects on target traits, which would most likely result from two scenarios, although this remains to be experimentally tested in future studies: (1) SVs cause loss of function mutants on target genes once occurred in their genic regions and (2) SVs cause dramatically changed expression patterns of target genes once occurred in the gene promoter regions. It should be noted that these are speculative interpretations based on previously reported cases of SV effects in other studies; direct evidence from the present study is not available to support either scenario. If so, SVs would have a major advantage in QTL cloning.

Indeed, an important QTL, *qLTG3‐1*, we found a deletion in the coding region of an Os03g0103300 gene that caused a loss of function (LOF) mutant, which was associated with significantly decreased LTG. Haplotype analysis results showed that almost all *Xian* accessions from low‐latitude regions with high LTG carried the NSV allele. On the other hand, approximately 24.1% of *Geng* accessions from the high‐latitude regions had low LTG and carried the deletion in this gene. Although S219970 exhibited the strongest association signal among the promoter SNPs of *qLTG3‐1*, and the haplotype comparison between Hap2 and Hap3 suggested a potential regulatory role, these lines of evidence are derived solely from association analyses and cannot establish a causal relationship between S219970 and LTG. On the one hand, Hap2 and Hap3 reside within a broader genomic region that may contain other unknown variants in linkage disequilibrium with S219970. On the other hand, as a putative promoter SNP, its effect on Os03g0103300 expression has not been directly verified. Therefore, S219970 should currently be regarded as a candidate SNP worthy of further investigation, rather than a confirmed functional variant. Future work is needed to clarify its independent genetic effect and molecular mechanism through direct approaches such as constructing near‐isogenic lines (NILs), promoter activity reporter assays, or CRISPR/Cas9‐mediated site‐directed base substitution. Nevertheless, at the *qLTG3‐1* locus, our novelty primarily lies in the systematic detection of SVs and the comprehensive haplotype analysis integrating promoter SNPs and coding region SVs, rather than the initial discovery of this gene.

Because of their high latitude and longitude distributions, *Geng* rice accessions are expected to have stronger cold tolerance than *Xian* rice accessions at both seedling and reproductive stages (Cheng et al., 2007). However, this was not always true at the germination stage. In one report, no significant difference in LTG between *Xian* and *Geng* accessions was found (Baruah et al., 2009), while in several other studies, *Xian* accessions were reported to have higher LTG than *Geng* accessions (Wang et al., 2011; Yang et al., 2020). In this study, a huge difference in LTG was observed between accessions of the two subspecies. *Xian* accessions exhibited average higher germination and seedling rates than those of *Geng* accessions in all four temperatures, though significant interactions were found between temperatures and subspecies. We found that seeds of *Xian* accessions tended to germinate faster than those of *Geng* accessions. The enrichment of high‐LTG haplotypes in low‐latitude regions, while seemingly counterintuitive from a simple cold tolerance perspective, can be explained by considering that LTG primarily reflects germination vigor rather than tolerance to persistent freezing. Early‐season rice in low‐latitude areas frequently encounters transient cold spells at sowing, favoring rapid germination under suboptimal temperatures. The non‐deletion allele represents the ancestral state, whereas the deletion variant emerged during the northward expansion of Geng rice and became fixed in high‐latitude populations, likely through hitchhiking with linked variants conferring reproductive‐stage cold tolerance or enhanced dormancy. These findings were consistent with the observed negative correlation between the latitude distribution of the 403 rice accessions and their LTG levels. The low‐latitude accessions exhibited the higher LTG than the higher latitude accessions, and vice versa. It should be noted that the negative correlation between origin latitude and LTG across the entire panel is partially confounded by subspecies identity, as high‐latitude accessions are predominantly *Geng* and low‐latitude ones are mainly *Xian*. However, stratified analysis within *Xian* revealed a significant positive correlation between latitude and GT50, indicating that latitude exerts an independent effect on LTG beyond subspecies differentiation. The lack of a similar trend within *Geng* likely reflects its narrower latitudinal range and strong domestication bottlenecks. Thus, the observed *Xian–Geng* difference in LTG reflects both subspecies‐specific genetic backgrounds and adaptive responses to latitudinal climatic gradients.

Our results indicated that LTG in panels *Xian* and *Geng* is under largely different genetic controls, reflected by the fact that few LTG QTL identified in panels *Xian* and *Geng* were common. Nevertheless, our results were highly consistent with previous reports in that at least 41.1% QTL/regions identified in this study, including six cloned LTG genes, *qLTG3‐1*, *OsSAP16*, *OsCAT2*, *OsABI5*, *OsSnRK1.1*, and *OsMYB30*, co‐localized in the same or overlapping regions that harbor QTL/genes associated with LTG reported previously (Tables S6 and S7). These novel QTL/regions with a fine resolution interval can be further functionally exploited to identify promising candidate genes. By combining GWAS and RNA‐seq results, 25 promising candidate genes of 11 important loci for LTG were identified. Significant LTG differences were found among different haplotypes at each of the candidate genes. The *Xian* panel, particularly accessions from low‐latitude areas, contains abundant high‐LTG alleles at the QTL candidate genes, whereas the *Geng* panel contains many low‐LTG alleles at the identified QTL candidate genes that were mainly distributed in the higher latitude regions. Thus, our results led us to the conclusion that the *Xian*–*Geng* differentiation in LTG is largely reflected by different QTL/genes controlling LTG and partially by allelic differences for high‐ and low‐LTG at many gene loci. The identification of 43 favorable alleles for high‐LTG at 25 candidate genes, once confirmed, would provide useful information for improving rice LTG by gene/allele pyramiding through various molecular breeding strategies. However, it should be noted that the observed correlations between gene PAVs and subspecific identities do not necessarily imply a causative role in driving *Xian–Geng* differentiation. Without direct evidence of ancestral allele states, which would require systematic analysis of wild rice accessions, and without knowledge of the temporal order of PAV emergence relative to the *Xian–Geng* split, the current data support only correlative inferences. The predominance of certain PAVs in one subspecies could equally result from founder effects, genetic drift, or selection on linked variants during domestication and subspecific divergence. Thus, while the PAV patterns documented here provide useful markers for subspecific differentiation, their role as drivers of the differentiation remains speculative and warrants further investigation via broader wild rice comparisons and functional characterization of the affected genes.

In addition to the utility of the favorable LTG haplotypes identified in this study for genetic improvement of LTG, excessive enhancement of LTG often carries the risk of increased susceptibility to pre‐harvest sprouting (PHS). Among the candidate genes we identified, *OsABI5* (corresponding to the locus *qLT1k*) is involved in the abscisic acid (ABA) signaling pathway and is known to regulate both seed dormancy and germination (Li et al., 2021). This functional overlap suggests that the genetic control of LTG and seed dormancy may be partially intertwined. However, all LTG phenotyping in this study was conducted under controlled growth chamber conditions. Therefore, before the high‐LTG alleles identified here can be confidently deployed in breeding programs, two lines of validation are required: field‐based assessment of PHS resistance for materials carrying these haplotypes and comprehensive dormancy assays to clarify the temperature specificity of the LTG enhancement. Thus, our current results should be viewed as identifying promising genetic resources for LTG improvement, and any practical breeding application must integrate PHS resistance as a key selection criterion alongside LTG performance. Beyond these practical considerations for breeding, the functional evidence obtained from gene editing also warrants a methodological distinction from the haplotype‐based association analyses. Gene‐editing experiments demonstrated that Os11g0438000 functions as a positive regulator of LTG in rice, as its LOF in the NIP background significantly impaired seed germination and seedling establishment under low‐temperature stress. This conclusion differs from the haplotype association analysis, in that the latter primarily revealed correlations between natural allelic variants at this locus and LTG phenotypic variation. Nevertheless, functional validation by gene editing and association analysis of natural variation provides converging evidence from different perspectives for the involvement of Os11g0438000 in LTG regulation.

## MATERIALS AND METHODS

### Plant material

In this study, a collection of 403 rice accessions from 26 countries was used to assess LTG, including 254 accessions selected from the 3010 Rice Genome Project (3KRGP) (Wang et al., 2018) and 149 accessions used in this study. The detailed information is listed in Table S1. All accessions were grown at the experimental station of Anhui Agricultural University in Sanya (18.24 N, 109.50E), China. Seeds of the *qLG11d* knock out (KO) lines generated via CRISPR/Cas9 gene‐editing technology, with Nipponbare (NIP) serving as the wild‐type (WT) genetic background. Each line was planted in two rows, with eight plants per row and a spacing of 20.0 × 25.0 cm, using a randomized complete block design.

### Phenotyping of the 403 rice accessions for LTG‐related traits

To make sure the harvested seeds of all 403 accessions to have uniform maturity, each of the 403 accessions were grown in a two‐three row plot under the normal irrigated conditions in the experimental farm of Anhui Agricultural University in Sanya (December – May). At the maturity, approximately 35–40 days after flowering, at least five plants in the middle of two rows of each accession were harvested. The pooled seeds were dried in an oven at 43°C for 3 days to break seed dormancy as described previously (Li et al., 2011; Zhang et al., 2020). Thirty seeds from each line were placed in a Petri dish with two sheets of filter paper, and 15 mL of distilled water was added for germination testing, which was performed in the growth chamber (Youke, Hefei, China) (Zhang et al., 2020). A seed was considered germinated when the length of its radicle was greater than 2 mm and a seedling was considered established when its root length reached the seed length and its shoot length was half of the seed length, as described previously (He et al., 2019; Yuan et al., 2019). Germination testing was performed on the 403 rice accessions under four temperatures: three low‐temperature conditions (13°C, 15°C, and 18°C) and one moderate temperature (21°C), with two to three independent replicates. The germinated seeds and seedlings in each Petri dish were counted every day for 17 consecutive days (from 1 day to 17 days) under the 13°C treatment, 13 consecutive days (from 1 day to 13 days) under the 15°C treatment, 9 consecutive days (from 1 day to 9 days) under the 18°C treatment, and 7 consecutive days (from 1 day to 7 days) under the 21°C treatment. Then, all four temperature treatments in the growth chamber were increased to 30°C for recovery with 4 days (Yang et al., 2020), and then the final germinated seeds and seedlings in each Petri dish were counted.

### Evaluation of parameters for LTG

A total of 39 seed germination and seedling related parameters were used to assess LTG. The detailed information and explanation of these parameters used in this study are listed in Table S2. These parameters included the area under germination and seedling percentage curves (GUC and SUC), the time (hours) required to reach 10% (GT10 and ST10), 50% (GT50 and ST50), 25–75% germination (GU) and seedling (SU) of each accession, and the time (hours) required to reach the average germination and seedling percentage (GMGT and SMGT), which were calculated using package GERMINATOR (Joosen et al., 2010). The low‐temperature germination rate (LT‐GR) and recovery germination rate (RT‐GR) were calculated as the cumulative numbers of germinated seeds during cold treatment and after subsequent recovery at 30°C, respectively, each divided by 30 seeds and expressed as percentages. The low‐temperature germinability index (LTG%) was defined as the ratio LT‐GR / RT‐GR × 100%, reflecting the proportion of viable seeds capable of germinating under cold conditions and thus correcting for differences in seed vigor among accessions. The seedling‐based index (LTS%) was calculated analogously using the corresponding seedling rates (LT‐SR and RT‐SR) (Yang et al., 2020). The mean values of the calculated parameters from the two replicates of each accession were used for data analysis. Pearson correlation coefficients (PCC) were calculated to assess the relationships between latitude and each LTG‐related parameter. To compare the mean correlation coefficients among the whole population, *Xian*, and *Geng* subgroups, Fisher's z transformation was applied to approximate normality, and Duncan's multiple range test was used for multiple comparisons.

### SNP calling and SV detection

The FASTQ dataset of the overlapped 3 K rice accessions was downloaded from the European Nucleotide Archive (https://www.ebi.ac.uk/ena/browser/home), and the remaining accessions used in this study were sequenced at China National Genebank (https://www.cngb.org/). The high‐quality reads were mapped to *Geng* Nipponbare reference genome using BWA (v0.7.15) (Li & Durbin, 2009) with the default parameters, and filtered by SAMtools (Li et al., 2009). Picard package (v1.87) (http://broadinstitute.github.io/picard/) was used to filter the duplicated reads. SNP calling of the mapping data was carried out by GATK (McKenna et al., 2010). VCFtools (Danecek et al., 2011) was used to filter the SNPs. Delly (v1.1.6) (https://github.com/dellytools/delly) was used for SV detection. Five types of SVs were detected using the default parameters of Delly, including deletions (Dels), insertions (INSs), duplications (DUPs), inversions (INVs), and break‐ends (BNDs). To improve the accuracy of SV detection, the results were filtered using the following criteria: the SVs smaller than 10‐bp or within centromeres, and contigs <200 kb were excluded.

### Population structure analysis

The high‐quality SNPs and SVs were used to construct neighbor‐joining (NJ) trees with 1000 bootstrap replicates in software FastME (version 2.15) (http://www.atgc‐montpellier.fr/fastme/) and iTOL (https://itol.embl.de). Principal component analysis (PCA) based on the SNPs and SVs was performed using PLINK (Purcell et al., 2007) to estimate the subpopulation classes. The admixture software (version 1.3.0) (dalexander.github.io) was used to perform population structure analysis. Pairwise *r* ^2^ of high‐quality genome‐wide SNPs (MAF ≥0.05) was calculated with PopLDdecay (https://github.com/BGI‐shenzhen/PopLDdecay). LD decay distance was defined as the physical distance at which *r* ^2^ drops to half of its maximum value.

### GWAS of LTG based on SNPs and SVs

The LTG parameters were used as phenotypic input datasets to perform GWAS using SNPs and SVs in the whole, *Xian*, and *Geng* panels, respectively. GWAS with the mixed linear model (MLM) was performed using the “rMVP” package (Yin et al., 2021). After filtering out low‐quality SNPs with minor allele frequencies (MAF) ≤0.05 and missing rates ≥20%, a total of 3.2 M SNPs for the whole, 2.6 M SNPs for panel *Geng*, and 3.0 M SNPs for panel *Xian* were identified. To improve the accuracy of SVs, variants were filtered with MAF <0.05. To determine the significance thresholds in QTL detection, the effective SNP and SV number in each panel was calculated by software GEC (Li et al., 2012) using the total detected SNPs and SVs, which were used to replace the total number of SNPs and SVs. For the SNPs, the resultant thresholds (0.01/EN) for the whole, *Xian*, and *Geng* panels were 1.4 × 10^−8^, 1.1 × 10^−8^, and 1.8 × 10^−8^, respectively. The SNPs in such a signal region were merged into one unique block, and the peak SNP with the lowest *P*‐value in the region was considered as the lead SNP (Chen et al., 2014). Similarly stringent thresholds (0.001/EN) of 3.7 × 10^−8^, 3.5 × 10^−8^, and 4.9 × 10^−8^ were used for detecting QTL in panels whole, *Xian*, and *Geng* according to the effective SVs in GEC. Due to the smaller population size of the *Geng* accessions (*n* = 121), a less stringent threshold of *P* < 1.0 × 10^−6^ was used as the reference threshold for QTL detection in this study.

### RNA extraction and RNA‐seq analysis

Four high‐LTG accessions (two *Xian* accessions: YH72 and CA; and two *Geng* accessions: GM2 and HD502) and four low‐LTG accessions (two *Xian* accessions: TP3 and F88; and two *Geng* accessions: SG5 and NG47) were selected to conduct RNA transcriptome sequencing (RNA‐seq). The embryo sampling was done at 36 h after imbibition in a normal temperature (as check), and the RNA sampling were obtained at a low‐temperature treatment (13°C) for 4 h (CT4) and 8 h (CT4), with three biological replications. Total RNA was extracted using HP Plant RNA kit (Omega, Atlanta, GA) according to the manufacturer's instructions. RNA‐seq was performed on NovaSeq 6000 platform (Illumina) with paired‐end 150 (PE150) and PE150 paired‐end reads were generated. Clean reads were mapped to the *Geng* NIP reference genome using HISAT2. Differentially expressed genes (DEGs) were identified by two criteria of |log2FoldChange ≥1.0| and *P*‐value <0.05 in DESeq (1.20.0).

### Candidate gene identification and favorable allele mining

Positional candidate genes for each of the important QTL were searched (according to their LD results) surrounding the peak SNPs in the corresponding QTL. Annotations of genes located in a QTL were obtained from the Nipponbare Rice Genome Annotation Project Database (http://rice.plantbiology.msu.edu/) and RAP‐db (https://rapdb.dna.affrc.go.jp/). The candidate gene(s) were determined by the following steps: (1) all annotated genes were obtained, and then the genes were selected after the removal of transposons/retrotransposons or hypothetical proteins, and genes with almost no expression in the seeds or embryo; (2) the common DEGs from CT4 and CT8 located in QTL that were obtained from the RNA‐seq results; (3) all significant SNPs that locate in the 1‐kb promoter regions and gene coding regions (ignored non‐synonymous SNPs) in DEG were used for identifying haplotypes, and then for determining their phenotypic effects on the LTG parameters by comparing the phenotypic effects of different major haplotypes (accessions >10). Candidate genes were assigned by testing significant differences for corresponding parameters among major haplotypes for each major QTL by Duncan tests (*P* < 0.05); (4) when major QTL containing a cloned gene for LTG, the cloned gene was considered as the important candidate gene of the target QTL; (5) favorable alleles (haplotypes constructed using SNPs in the gene CDS and promoter regions) of the candidate were designated as haplotypes with significant increasing effects for LTG.

### Detection of candidate gene PAV–LTG associations

Two sets of gene PAV genotypic data were used in this study, including 453 rice accessions sequenced with NGS (Wang et al., 2018) and 110 rice accessions sequenced with third‐generation sequencing (TGS) technologies (Zhang et al., 2022). After ignoring 24 admix accessions, 539 accessions (six Wild, 359 *Xian*, 103 *Geng*, 48 Aus, and 23 Bas accessions) were used for further analysis. The phenotypic data for LTG of the additional 84 TGS accessions were performed in T15. Wilcoxon rank‐sum test was used to detect gene PAV–LTG associations (Zhang et al., 2022) in R (https://www.r‐project.org/). *P*‐values were adjusted using a threshold of 0.005 for claiming a significant gene PAV–LTG association.

### Population genetic differentiation analysis

Because the *Xian–Geng* differentiation represents the maximum direction of the genomic diversity in the 403 accessions and our previous data also showed highly significant differences in LTG between *Xian* and *Geng* subspecies, we estimated three population genetic parameters, including population differentiation statistics (FST), nucleotide diversity (π), and Tajima's D across the whole rice genome to determine the genomic regions with strong *Xian–Geng* differentiation using software PopGenome with 100‐kb windows and 10‐kb steps (Pfeifer et al., 2014). Sliding windows with the top 5% FST values were identified as the divergent windows that differentiate *Xian* and *Geng*. Adjacent divergent windows were merged into a single divergent region (DR), and the average value of all steps in a single DR was regarded as the FST value in a DR. DRs overlapped with the identified LTG QTL in this study, the QTL detected by linkage analysis, and all cloned genes for LTG reported previously were defined as the DRs related to LTG (DR_LGs). DRs with average Tajima's D < −1 and Tajima's D > 1 in corresponding populations, which represent that DRs had gone through positive or negative selection for LTG during evolution, were used as criteria to screen the DRs.

### Genomic selection model construction using SNP and SV datasets

Model training and analysis were performed using the MFMGP software (Multiple Machine Learning Fusion Models Genomic Prediction) (https://mfmgp.bioaibp.com/#/Breeding/MFMGP/) with both SNP and SV genotypic datasets, which were constructed by integrating four machine learning (ML) algorithms (XGBoost, LightGBM, Extra Trees, and HGBoost algorithms). Model performance was evaluated via 10‐fold cross‐validation (10‐fold CV), and two key metrics, the Pearson correlation coefficient (PCC) and root mean squared error (RMSE), were calculated in each fold. PCC was used to measure the linear correlation between predicted and observed values, ranging from −1 to 1, and its magnitude reflects the model's prediction accuracy: a value of 1 indicates a perfect positive linear correlation, −1 indicates a perfect negative linear correlation, and 0 indicates no linear correlation. RMSE is the square root of the mean squared error (MSE) and quantifies the standard deviation of the differences between predicted and observed values. The average values of the two metrics across the 10‐fold CV were calculated.

## Author Contributions

CZ, LM, WW, and ZL designed and conceived research; LM, YS, WC, XH, and TY performed phenotyping; LP and TF performed genotyping analysis; YS, YL, and LZ analyzed data; ZP, YS, LM, and WS wrote the paper; LB developed populations; All authors read and approved the final manuscript.

## Conflict of Interest Statement

The authors state that they have no conflict of interest.

## Supporting information


**Table S1.** The information list of the 403 rice accessions.
**Table S2**. The parameters used in this study to assess low‐temperature germinability of rice.
**Table S3**. The numbers of five types of structural variations (SVs) and their genomic distributions identified in the 403 rice genomes.
**Table S4**. Phenotypic performances of 403 rice accessions for traits contributing to low‐temperature germinability and seedling under four temperature conditions.
**Table S5**. Analysis of variance (ANOVA) of five traits contributing to low‐temperature germinability measured in 403 rice accessions under four low‐temperature conditions.
**Table S6**. The QTLs associated with low‐temperature germinability identified using SNP dataset.
**Table S7a**. The significant SVs associated with low‐temperature germinability using SV dataset.
**Table S7b**. The QTLs associated with low‐temperature germinability identified using SV dataset.
**Table S8**. The important QTLs for LTG identified by SNP‐ and SV‐based GWAS.
**Table S9**. Germination parameters and early seedling growth of wild‐type (WT) and knockout mutants (KO1, KO2) under different temperature treatments.
**Table S10**. The candidate genes of important LTG QTL and their favorable alleles contributing to high LTG.
**Table S11**. Pearson correlation coefficients between latitude and low‐temperature germination‐related parameters in Xian and Geng subpopulations under different temperatures.
**Table S12**. Sixty‐one genomic regions differentiated between populations Geng and Xian.
**Table S13**. Population genetic parameters of 29 divergent regions to low‐temperature germinability between Xian and Geng.
**Table S14**. Genomic regions differentiated within the Xian subspecies.
**Table S15**. Population genetic parameters of 37 divergent regions to low‐temperature germinability within the Xian subspecies.
**Table S16**. The common genomic divergent regions (DRs) between the Xian/Xian and Xian/Geng.
**Table S17**. Prediction accuracy of genomic prediction models under within‐ and cross‐subpopulation validation for low‐temperature germination using SV dataset.

**Figure S1.** Distribution of single‐nucleotide polymorphisms (SNPs) and structural variants (SVs) across the whole rice genome. The SNPs (A) and SVs (B) generated from the Nipponbare reference genome, with each row representing one chromosome and each vertical bar of individual chromosomes denoting SNP in every 1‐Mb interval and SVs in every 500‐kb interval. Different colors on the bottom right show the density of SNPs and SVs.
**Figure S2**. Genotype frequencies at each SV in the 403 rice accessions. The cyan and orange colors indicate homozygous SV (accessions possessing homozygous alternative allele) and NSV (accessions with homozygous reference (Nipponbare) type of allele) genotype, respectively. The *x*‐axis represents the physical location of the SV markers along each numbered chromosome. The *y*‐axis represents the genotype frequency.
**Figure S3**. Average frequencies of NSVs (accessions with homozygous reference (Nipponbare) type of allele) and SVs (accessions possessing homozygous alternative allele) in the whole, *Xian*, and *Geng* panels, respectively.
**Figure S4**. Analysis of the quantity, frequency distribution, and cumulative proportion of *Xian*‐specific and *Geng*‐specific structural variations (SVs).
**Figure S5**. Low‐temperature germinability (LTG) of 403 rice accessions across different temperatures. (A–D) Seed germination (G_7d_) of 403 rice accessions in 13°C (T13), 15°C (T15), 18°C (T18), and 21°C (T21). (E–H) Differences in LTG between *Xian* and *Geng* subpopulations.
**Figure S6**. Differences in temperature germinability (LTG) between *Xian* and *Geng* subpopulations across different environments. The red and blue box denote *Geng* and *Xian* subpopulations, respectively. Time required to reach 10% germination (GT10); time required to reach 50% germination (GT50); time required to reach 10% seedling (ST10); time required to reach 50% seedling (ST50); germination index (GI); seedling index (SI).
**Figure S7**. The common QTL for temperature germinability (LTG) identified by different temperatures.
**Figure S8**. The germination performance of a quantitative trait locus (QTL) (*qLT3a/qLTv3a*, quantitative trait locus for low‐temperature germinability on chromosome 3a, detected by both SNP–GWAS and SV–GWAS) that was detected by SNP‐ and SV‐based GWAS. (A–D) Phenotype differences between the SV and NSV groups in the whole panel. (B) Phenotype differences between the SV and NSV groups in the *Geng* panel. Double asterisks denote significant differences at *P* < 0.01.
**Figure S9**. Geographic distribution of the four major haplotypes (Hap1–Hap4) of *qLTG3‐1* across 403 accessions.

## Data Availability

The data that support the findings of this study are available on request from the corresponding author. The data are not publicly available due to privacy or ethical restrictions.
