# Supplementary material for: Genetic architecture of low‐temperature germinability in rice revealed by integrated maps of SNPs and structural variation
Source: Plant J. 2026 Oct 27;128(2):e71184. doi: 10.1111/tpj.71184 (PMC13711015; doi:10.1111/tpj.71184)
Supplement: Supplementary file 1 [file TPJ-128-0-s001.docx]

Figure S1. Distribution of single nucleotide polymorphisms (SNPs) and structural variants (SVs) across the whole rice genome. The SNPs (A) and SVs (B) generated from the Nipponbare reference genome, with each row representing one chromosome and each vertical bar of individual chromosomes denoting SNP in every 1-Mb interval and SVs in every 500-kb interval. Different colors on the bottom right show the density of SNPs and SVs.

Figure S2. Genotype frequencies at each SV in the 403 rice accessions. The cyan and orange colors indicate homozygous SV (accessions possessing homozygous alternative allele) and NSV (accessions with homozygous reference (Nipponbare) type of allele) genotype, respectively. The *x*-axis represents the physical location of the SV markers along each numbered chromosome. The *y*-axis represents the genotype frequency.

Figure S3. Average frequencies of NSVs (accessions with homozygous reference (Nipponbare) type of allele) and SVs (accessions possessing homozygous alternative allele) in the whole, *Xian*, and *Geng* panels, respectively.

Figure S4. Analysis of the quantity, frequency distribution and cumulative proportion of *Xian*-specific and *Geng*-specific structural variations (SVs).

Figure S5**.** Low temperature germinability (LTG) of 403 rice accessions across different temperatures. (A-D) Seed germination (G_7d_) of 403 rice accessions in 13°C (T13), 15°C (T15), 18°C (T18), and 21°C (T21). (E-H) Differences in LTG between *Xian* and *Geng* subpopulations.

Figure S6. Differences in temperature germinability (LTG) between *Xian* and *Geng* subpopulations across different environments. The red and blue box denote *Geng* and *Xian* subpopulations, respectively. Time required to reach 10% germination (GT10); time required to reach 50% germination (GT50); time required to reach 10% seedling (ST10); time required to reach 50% seedling (ST50); germination index (GI); seedling index (SI).

Figure S7. The common QTL for temperature germinability (LTG) identified by different temperatures.

Figure S8. The germination performance of a quantitative trait locus (QTL) (*qLT3a/qLTv3a*, quantitative trait locus for low-temperature germinability on chromosome 3a, detected by both SNP-GWAS and SV-GWAS) that was detected by SNP- and SV-based GWAS. (A-D) Phenotype differences between the SV and NSV groups in the whole panel. (B) Phenotype differences between the SV and NSV groups in the *Geng* panel. Double asterisks denote significant differences at *P* < 0.01.

Figure S9. Geographic distribution of the four major haplotypes (Hap1-Hap4) of *qLTG3-1* across 403 accessions.
